# Announcement as effort on topological spaces

**DOI:** 10.1007/s11229-017-1592-8

**Published:** 2017-10-29

**Authors:** Hans van Ditmarsch, Sophia Knight, Aybüke Özgün

**Affiliations:** 1 LORIA, CNRS, Université de Lorraine, Nancy, France; 2 Uppsala University, Uppsala, Sweden; 3 ILLC, University of Amsterdam, Amsterdam, The Netherlands

**Keywords:** Topology, Subset space logic, Dynamic epistemic logic, Arbitrary (public)announcements

## Abstract

We propose a multi-agent logic of knowledge, public announcements and arbitrary announcements, interpreted on topological spaces in the style of subset space semantics. The arbitrary announcement modality functions similarly to the effort modality in subset space logics, however, it comes with intuitive and semantic differences. We provide axiomatizations for three logics based on this setting, with *S*5 knowledge modality, and demonstrate their completeness. We moreover consider the weaker axiomatizations of three logics with *S*4 type of knowledge and prove soundness and completeness results for these systems.

## Introduction

Moss and Parikh (1992) introduce a bi-modal logic with language

$$\begin{aligned} \varphi :{:=} p \ | \ \lnot \varphi \ | \ \varphi \wedge \varphi \ | \ K\varphi \ | \ \Box \varphi , \end{aligned}$$

called subset space logic (SSL), in order to formalize reasoning about sets and points together in a particular modal system. The main interest in their investigation lies in spatial structures such as topological spaces, and using modal logic and the techniques behind it for spatial reasoning; however, they also have a strong motivation from epistemic logic. While the modality *K* is interpreted as knowledge, $\Box$ is intended to capture the notion of *effort*, i.e., any action that results in an increase in knowledge; such as measurement, computation, approximation or even an announcement. While the shape of effort may vary depending on the context and the source of information, one fundamental and common constituent is taken to be *observation* (Moss and Parikh 1992). Such a rich epistemic setting capturing observational effort and knowledge therefore demands well-equipped models in order to be able to represent the aforementioned concepts. Moss and Parikh (1992) therefore *propose subset space semantics* for their logic. A subset space is defined to be a pair $(X, {\mathcal {O}})$, where *X* is a non-empty set of states and ${\mathcal {O}}$ is a collection of subsets of *X* (not necessarily a topology, however topological spaces constitute a particular case of subset spaces).^1^ The elements of ${\mathcal {O}}$ are considered as *possible observations* or *possible observation sets*, and the formulas are interpreted not only with respect to the actual state, but with respect to pairs of the form (*x*, *U*), where $x\in U\in {\mathcal {O}}$: while *x* represents the way the actual state of affairs is, the *neighbourhoodU* with $x\in U\in {\mathcal {O}}$ is taken to be a truthful observation that can be made about the actual state *x* (Moss and Parikh 1992). According to subset space semantics, given a pair (*x*, *U*), the modality *K* quantifies over the elements of *U*, whereas $\Box$ quantifies over all subsets of *U* in ${\mathcal {O}}$ that include the actual world *x*. Therefore, while knowledge is interpreted ‘locally’ in a given turthful observation set *U*, effort is read as *neighbourhood-shrinking* where more effort corresponds to a smaller neighbourhood, i.e., a more refined truthful observation, thus, a possible increase in knowledge. The schema $\Diamond K\varphi$ states that after some effort the agent comes to know $\varphi$, where effort can be in the form of measurement, computation, approximation (Moss and Parikh 1992; Dabrowski et al. 1996; Parikh et al. 2007; Başkent 2012), or announcement (Plaza 1989; Balbiani et al. 2008; van Ditmarsch et al. 2014).

The epistemic motivation behind the subset space semantics and the dynamic nature of the effort modality suggests a link between SSL and dynamic epistemic logic, in particular dynamics known as public announcement, as also noted by Georgatos (2011), and studied in Başkent (2007, 2012), Balbiani et al. (2013), Wáng and Ågotnes (2013b), Bjorndahl (2017). Başkent (2007, 2012) and Balbiani et al. (2013) propose modelling public announcements on subset spaces by deleting the states or the neighbourhoods falsifying the announcement. This dynamic epistemic method is not in the spirit of the effort modality: dynamic epistemic actions result in global model change, whereas the effort modality results in local neighbourhood shrinking without leading to any change in the model under consideration. Hence, it is natural to search for a ‘neighbourhood-shrinking-like’ interpretation of public announcements on subset spaces. Wáng and Ågotnes (2013b) first proposed semantics for public announcements on subset spaces in the style of the effort modality, although the subset spaces used here are not necessarily topological spaces. Bjorndahl (2017) then proposed a revised version of the semantics of Wáng and Ågotnes (2013b). In contrast to the aforementioned proposals, Bjorndahl (2017) uses models based on topological spaces to interpret knowledge and information change via public announcements. He considers the language

$$\begin{aligned} \varphi :{:=} p \ | \ \lnot \varphi \ | \ \varphi \wedge \varphi \ | \ K\varphi \ | \ { int} (\varphi ) \ | \ [\varphi ]\varphi , \end{aligned}$$

where ${ int} (\varphi )$, roughly speaking, means ‘$\varphi$ is true and can be announced’ and where $[\varphi ]\psi$ means ‘after public announcement of $\varphi$, $\psi$ (is true).’ More precisely, in this topological framework, the novel modality ${ int} (\varphi )$ plays the role of the precondition for the public announcement of $\varphi$ and it is interpreted as the interior operator on topological spaces. The precondition ${ int} (\varphi )$ is stronger than $\varphi$ only being true: it moreover states that $\varphi$*is supported by a truthful observation* (as opposed to the standard precondition for the public announcements, that is, the announced formula only being true, see e.g., van Ditmarsch et al. 2007, 2015 for a survey). This modality is also an important part of our current work and it will be analysed in detail, both syntactically and semantically, in later sections.

Balbiani et al. (2008) introduce a logic to quantify over announcements in the setting of epistemic logic based on the language (with single-agent version here)

$$\begin{aligned} \varphi :{:=} p \ | \ \lnot \varphi \ | \ \varphi \wedge \varphi \ | \ K \varphi \ | \ [\varphi ]\varphi \ | \ \Box \varphi . \end{aligned}$$

In this case, unlike the above SSL setting where $\Box \varphi$ is read as ‘after any *effort*, $\varphi$ (is true)’, the so-called arbitrary announcement modality $\Box \varphi$ means ‘after any *announcement*, $\varphi$ (is true)’. It therefore quantifies over only *epistemically definable* subsets ($\Box$-free formulas of the language) of a given model. In this case, $\Diamond K \varphi$ again means that the agent comes to know $\varphi$, but in the interpretation that there is a formula $\psi$ such that after announcing it the agent knows $\varphi$. What becomes true or known by an agent after an announcement can be expressed in this language without explicit reference to the announced formula.

Clearly, the meaning of the effort $\Box$ modality (of Moss and Parikh 1992) and of the arbitrary announcement $\Box$ modality (of Balbiani et al. 2008) are related in motivation. In both cases, interpreting the modality requires quantification over sets. Subset-space-like semantics provides natural tools for this. van Ditmarsch et al. (2014) extended the proposal in Bjorndahl (2017) with an arbitrary announcement modality

$$\begin{aligned} \varphi :{:=} p \ | \ \lnot \varphi \ | \ \varphi \wedge \varphi \ | \ K\varphi \ | \ { int} (\varphi ) \ | \ [\varphi ]\varphi \ | \ \Box \varphi \end{aligned}$$

and provided topological semantics for the $$\Box $$ modality, and proved completeness for the corresponding single-agent logic $${ APAL}_{ int} $$. Baltag et al. (2017) later showed that, in spite of their different readings, the arbitrary announcement modality and the effort modality are equivalent in the single-agent case on topological spaces.

In this paper, we generalize the topological arbitrary announcement setting in van Ditmarsch et al. (2014) to a multi-agent setting, wherein the language becomes

$$\begin{aligned} \varphi :{:=} p \ | \ \lnot \varphi \ | \ \varphi \wedge \varphi \ | \ K_i\varphi \ | \ { int} (\varphi ) \ | \ [\varphi ]\varphi \ | \ \Box \varphi . \end{aligned}$$

The only difference with the previous language is that the knowledge operator now has an index: $K_i \varphi$ means that agent *i* knows $\varphi$. Multi-agent subset space logics have been investigated in Heinemann (2008, 2010), Başkent (2007) and Wáng and Ågotnes (2013a). There are some challenges with such a logic concerning the evaluation of higher-order knowledge. The general setup is for any finite number of agents, but to demonstrate the challenges, consider the case of two agents. If we extend the setup from the single agent case in the straightforward way, then for each of two agents *i* and *j* there is an open set and the semantic primitive becomes a triple $(x,U_i,U_j)$ instead of a pair (*x*, *U*). Now consider a formula like $K_i \hat{K}_j K_i p$, for ‘agent *i* knows that agent *j* considers possible that agent *i* knows proposition *p*’. If this is true for a triple $(x,U_i,U_j)$, then $\hat{K}_j K_i p$ must be true for any $y \in U_i$; but *y* may not be in $U_j$, in which case $(y,U_i,U_j)$ is not well-defined: we cannot interpret $\hat{K}_j K_i p$. Our solution to this dilemma is to consider neighbourhoods that are not only relative to each agent, as usual in multi-agent subset space logics, but that are also *relative to each state*. This amounts to, when shifting the viewpoint from *x* to $y \in U_i$, in $(x,U_i,U_j)$, we simultaneously have to shift the *neighbourhood* (and not merely the point in the actual neighbourhood) for the other agent. So we then go from $(x,U_i,U_j)$ to $(y,U_i,V_j)$, where $V_j$ may be different from $U_j$: $U_j$ represents *j*’s observation at *x* and $V_j$ represents *j*’s observation at *y*. Therefore, the neighbourhood shift from $U_j$ to $V_j$ does not mean a change of agent *j*’s observation at the actual state. While the tuple $(x, U_i, U_j)$ represents the actual state and the view points of both agents, the components $(y, V_j)$ of the latter tuple merely represents agent *j*’s epistemic state from agent *i*’s perspective at *y*, a possibly different state from the actual state *x*.

In order to define the evaluation neighbourhood for each agent with respect to the state in question, we employ a technique inspired by the standard neighbourhood semantics (Chellas 1980). We use a set of *neighbourhood functions*, determining the evaluation neighbourhood relative to both the given state and the corresponding agent. These functions need to be partial in order to render the semantics well-defined for the dynamic modalities in the system.

Using topological spaces enriched with a set of (partial) neighbourhood functions as models allows us to work with different notions of knowledge. In the standard (single-agent) SSL setting, as the knowledge modality quantifies over the elements of a fixed neighbourhood, the *S*5 type knowledge is inherent to the way the semantics defined. With our approach, however, the epistemic view of an agent changes according to the neighbourhood functions when the evaluation state changes, therefore, the valid properties of knowledge are determined by the constraints imposed on the neighbourhood functions. To this end, we work with both the *S*5 and *S*4 types of knowledge in this paper: while the former is the standard notion of knowledge in the subset space setting, the latter reveals a novel aspect of our approach, namely, the ability to capture different notions of knowledge.

In Sect. 2 we define the syntax, structures, and semantics of our multi-agent logic of arbitrary public announcements, $${ APAL}_{ int} $$, interpreted on topological spaces equipped with a set of neighbourhood functions. Without arbitrary announcements we get the logic $${ PAL}_{int}$$, and with neither arbitrary nor public announcements, the logic $${ EL}_{int}$$. In this section we also show some typical validities, and give two detailed examples. In Sect. 3 we give axiomatizations for the logics: $${ PAL}_{int}$$ extends $${ EL}_{int}$$ and $${ APAL}_{ int} $$ extends $${ PAL}_{int}$$. In Sect. 4 we demonstrate completeness for these logics. The completeness proof for the epistemic version of the logic, $${ EL}_{int}$$, is rather different from the completeness proof for the full logic $${ APAL}_{int}$$. Section 5 adapts the logics to the case of *S*4 knowledge. In Sect. 6 we compare our work to that of others, and then conclude.

**Prior work** This work should be seen as the journal version of the extended abstract presented in van Ditmarsch et al. (2015b). This journal version: contains more proof details, for example, in Propositions 14–18 and in Lemma 40; provides an additional extended example with a truly topological character (Sect. 2.6.2); uses another complexity measure in the completeness proof (for better integration with the ${ PAL}_{int}$ completeness proof); has a new Sect. 2.5 on the least topological model (given a set of neighbourhood functions); has a new Sect. 5 on *S*4 knowledge; and has a new Sect. 6.1 embedding single-agent into multi-agent topological semantics and vice versa.

## The logic $${ APAL}_{ int} $$

We define the syntax, structures, and semantics of our logic. From now on, $Prop$ is a countable set of propositional variables and ${\mathcal {A}}$ a finite and non-empty set of agents.

### Syntax

#### Definition 1

The language $\mathcal {L}_{{ APAL}_{ int} }$ is defined by

$$\begin{aligned} \varphi :{:=} p \ | \ \lnot \varphi \ | \ \varphi \wedge \varphi \ | \ K_i\varphi \ | \ { int} (\varphi ) \ | \ [\varphi ]\varphi \ | \ \Box \varphi \end{aligned}$$

where $$p\in Prop $$ and $$i\in {\mathcal {A}}$$. Abbreviations for the connectives $$\vee $$, $$\rightarrow $$ and $$\leftrightarrow $$ are standard, and $$\bot $$ is defined as abbreviation by $$p\wedge \lnot p$$. We employ $$\hat{K}_i\varphi $$ for $$\lnot K_i \lnot \varphi $$, and $$\Diamond \varphi $$ for $$\lnot \Box \lnot \varphi $$. We denote the non-modal part of $$\mathcal {L}_{{ APAL}_{ int} }$$ (without the modalities $$K_i$$, $${ int} $$, $$[\varphi ]$$ and $$\Box $$) by $${\mathcal {L}}_{Pl}$$, the part without $$\Box $$ by $${\mathcal {L}}_{{ PAL}_{ int} }$$, and the part without $$\Box $$ and $$[\varphi ]$$ by $${\mathcal {L}}_{{ EL}_{ int} }$$.

Necessity forms (Goldblatt 1982) allow us to select unique occurrences of a subformula in a given formula (unlike in uniform substitution). They will be used in the axiomatization (Sect. 3).

#### Definition 2

Let $\varphi \in {\mathcal {L}}_{{ APAL}_{ int} }$. The *necessity forms* are inductively defined as

$$\begin{aligned} \xi (\sharp ):{=} \sharp \ | \ \varphi \rightarrow \xi (\sharp ) \ | \ K_i\xi (\sharp ) \ | \ { int} (\xi (\sharp )) \ | \ [\varphi ]\xi (\sharp ). \end{aligned}$$

Each necessity form $\xi (\sharp )$ has a unique occurrence of $\sharp$. Given a necessity form $\xi (\sharp )$ and a formula $\varphi \in {\mathcal {L}}_{{ APAL}_{ int} }$, the formula obtained by replacing $\sharp$ by $\varphi$ is denoted by $\xi (\varphi )$.

In the Truth Lemma of the completeness proof (Lemma 40, Sect. 4) we need a complexity measure on formulas wherein, for example, $[\psi ]\varphi$ is less complex than $\Box \varphi$. Therefore, the subformula complexity of formulas does not suffice. The appropriate complexity measure is composed of a measure $S(\varphi )$ that is a weighted count of the number of symbols and a measure $d(\varphi )$ that counts the number of the $\Box$-modalities occurring in a formula.

#### Definition 3

The size $S(\varphi )$ of formula $\varphi \in {\mathcal {L}}_{{ APAL}_{ int} }$ is defined as:

$$\begin{aligned} S(p)= & {} 1,\\ S(\lnot \varphi )= & {} S(\varphi )+1,\\ S(\varphi \wedge \psi )= & {} S(\varphi )+S(\psi )+1,\\ S(K_i\varphi )= & {} S(\varphi )+1,\\ S({ int} (\varphi ) )= & {} S(\varphi )+1,\\ S([\varphi ]\psi )= & {} 4(S(\varphi )+4)S(\psi ),\\ S(\Box \varphi )= & {} S(\varphi )+1. \end{aligned}$$

The clauses for conjunction and public announcement in $$S(\varphi )$$ are different from the similar measure defined in Balbiani and van Ditmarsch (2015), and also different from the measure used in van Ditmarsch et al. (2015b). The measures used there are of course fine, however, we preferred a complexity measure that we could not only use in the completeness proof of $${ APAL}_{ int} $$ but also in the completeness proof of public announcement logic $${ PAL}_{ int} $$.

#### Definition 4

The $\Box$-depth $d(\varphi )$ of formula $\varphi \in {\mathcal {L}}_{{ APAL}_{ int} }$ is defined as:

$$\begin{aligned} d (p)= & {} 0,\\ d (\lnot \varphi )= & {} d (\varphi ),\\ d (\varphi \wedge \psi )= & {} max \{d (\varphi ), d (\psi )\},\\ d (K_i\varphi )= & {} d (\varphi ),\\ d ({ int} (\varphi ) )= & {} d (\varphi ),\\ d ([\varphi ]\psi )= & {} max \{d (\varphi ), d (\psi )\},\\ d (\Box \varphi )= & {} d (\varphi )+1 \end{aligned}$$

We now define three order relations on $\mathcal {L}_{{ APAL}_{ int} }$ based on the size and $\Box$-depth of the formulas.

#### Definition 5

For any $$\varphi , \psi \in {\mathcal {L}}_{{ APAL}_{ int} }$$,

- $$\varphi <^S \psi $$ iff $$S(\varphi )<S(\psi )$$
- $$\varphi <_d \psi $$ iff $$d (\varphi )<d (\psi )$$
- $\varphi <^S_d \psi$ iff (either $d (\varphi )<d (\psi )$, or $d (\varphi )=d (\psi )$ and $S(\varphi )< S(\psi )$)

We let ${ Sub}(\varphi )$ denote the set of subformulas of a given formula $\varphi$.

#### Lemma 6

For any $$\varphi , \psi \in {\mathcal {L}}_{{ APAL}_{ int} }$$,

1. $<^S,<_d, \ <^S_d$ are well-founded strict partial orders between formulas in ${\mathcal {L}}_{{ APAL}_{ int} }$,
2. if $\varphi \in { Sub}(\psi )$ and $\varphi$ is not $\psi$, then $\varphi <^S_d \psi$,
3. $${ int} (\varphi ) <^S_d [\varphi ]\psi $$,
4. $$\varphi \in {\mathcal {L}}_{{ PAL}_{ int} }$$ iff $$d (\varphi )=0$$,
5. $$\varphi \in {\mathcal {L}}_{{ PAL}_{ int} }$$ implies $$[\varphi ]\psi <^S_d \Box \psi $$.

#### Lemma 7

For any $\varphi , \psi , \chi \in {\mathcal {L}}_{{ APAL}_{ int} }$ and $i\in {\mathcal {A}}$,

1. $${ int} (\varphi ) \rightarrow p <^S_d [\varphi ]p$$,
2. $${ int} (\varphi ) \rightarrow \lnot [\varphi ]\psi <^S_d [\varphi ]\lnot \psi $$,
3. $$ [\varphi ]\psi \wedge [\varphi ]\chi <^S_d [\varphi ](\psi \wedge \chi ) $$,
4. $${ int} (\varphi ) \rightarrow { int} ([\varphi ]\psi ) <^S_d [\varphi ]{ int} (\psi ) $$,
5. $${ int} (\varphi ) \rightarrow K_i [\varphi ]\psi <^S_d [\varphi ] K_i\psi $$,
6. $$[\lnot [\varphi ]\lnot { int} (\psi ) ]\chi <^S_d [\varphi ][\psi ]\chi $$.

#### Proof

We prove Lemmas 7.3, 7.4 and 7.6. The proofs for the other items follow similarly. We define $\varphi \rightarrow \psi$ as $\lnot (\varphi \wedge \lnot \psi )$, so that $S(\varphi \rightarrow \psi )= S(\varphi ) + S(\psi ) +3$.

(7.3) On the left-hand-side, we have $S([\varphi ]\psi \wedge [\varphi ]\chi ) =1 + 4(S(\varphi ) + 4)(S(\psi )+S(\chi ))$. However, $S([\varphi ](\psi \wedge \chi ))=4 (S(\varphi )+ 4)(1+ S(\psi )+S(\chi )) = 4 (S(\varphi )+4) +4(S(\varphi )+4)(S(\psi )+S(\chi ))$. Thus, $S([\varphi ]\psi \wedge [\varphi ]\chi )< S([\varphi ](\psi \wedge \chi ))$. Moreover, $d([\varphi ]\psi \wedge [\varphi ]\chi )= max\{d(\varphi ), d(\psi ), d(\chi )\} = d([\varphi ](\psi \wedge \chi ))$ (This is similar in the other items). Therefore, by Definition 5, we obtain $[\varphi ]\psi \wedge [\varphi ]\chi <^S_d [\varphi ](\psi \wedge \chi )$.
(7.4) On the left-hand-side, we have $S({ int} (\varphi ) \rightarrow { int} ([\varphi ]\psi ) ) = S({ int} (\varphi ) )+S({ int} ([\varphi ]\psi ) )+3 = 1+S(\varphi )+1+S([\varphi ]\psi )+3=5+S(\varphi )+4S(\varphi )S(\psi )+16S(\psi ).$ However, $S([\varphi ]{ int} (\psi ) )=4S((\varphi )+4)S({ int} (\psi ) )= 4S((\varphi )+4)(S(\psi )+1)= 16 + 4S(\varphi )+4S(\varphi )S(\psi )+16 S(\psi ).$ Therefore, $S({ int} (\varphi ) \rightarrow { int} ([\varphi ]\psi ) )<S([\varphi ]{ int} (\psi ) )$. As in case (7.3) the $\Box$-depth of both formulas is the same. Therefore, ${ int} (\varphi ) \rightarrow { int} ([\varphi ]\psi ) <^S_d [\varphi ]{ int} (\psi )$.
(7.6) By Definition 3, we have that $S([\lnot [\varphi ]\lnot { int} (\psi ) ]\chi )= 4(S(\lnot [\varphi ]\lnot { int} (\psi ) )+4)S(\chi )=4 (5+4(S(\varphi )+4)(2+S(\psi )))S(\chi )= 4S(\chi )(37+8S(\varphi )+16S(\psi )+4S(\varphi )S(\psi )).$ On the other hand, $S([\varphi ][\psi ]\chi ) = 4 (S(\varphi )+4) 4 (S(\psi )+4)S(\chi )=4S(\chi )(64+16S(\varphi )+16S(\psi )+4S(\varphi )S(\psi )).$ Thus, as for any $\chi \in \mathcal {L}_{{ APAL}_{ int} }$, $1 \le S(\chi )$, $S([\lnot [\varphi ]\lnot { int} (\psi ) ]\chi )< S([\varphi ][\psi ]\chi )$. Further, we observe that $d([\lnot [\varphi ]\lnot { int} (\psi ) ]\chi ) = \max \{d(\varphi ), d(\psi ), d(\chi )\} = d([\varphi ][\psi ]\chi )$. Therefore, $[\lnot [\varphi ]\lnot { int} (\psi ) ]\chi <^S_d [\varphi ][\psi ]\chi$.

$$\square $$

### Background on topology

In this section, we introduce the topological concepts that will be used throughout this paper. All the concepts in this section can be found in Dugundji (1966).

#### Definition 8

A *topological space* is a pair $(X, \tau )$, where *X* is a non-empty set and $\tau$ is a family of subsets of *X* containing *X* and $\emptyset$, and is closed under finite intersections and arbitrary unions.

The set *X* is called the *space*. The subsets of *X* belonging to $\tau$ are called *open sets* (or *opens*) in the space; the family $\tau$ of open subsets of *X* is also called a *topology* on *X*. If for some $x\in X$ and an open $U\subseteq X$ we have $x\in U$, we say that *U* is an *open neighborhood* of *x*.

A point *x* is called an *interior point* of a set $$A\subseteq X$$ if there is an open neighborhood *U* of *x* such that $$U\subseteq A$$. The set of all interior points of *A* is called the *interior* of *A* and denoted by $${ Int} (A) $$. We can then easily observe that for any $$A\subseteq X$$, $${ Int} (A) $$ is an open set and is indeed the largest open subset of *A*.

#### Definition 9

A family ${\mathcal {B}}\subseteq \tau$ is called a *base* for a topological space $(X, \tau )$ if every non-empty open subset of *X* can be written as a union of elements of ${\mathcal {B}}$.

We can also give an equivalent definition of an interior point by referring only to a base ${\mathcal {B}}$ for a topological space $(X, \tau )$: for any $A\subseteq X$, $x\in { Int} (A)$ if and only if there is an open set $U\in {\mathcal {B}}$ such that $x\in U$ and $U\subseteq A$.

Given any family $\Sigma =\{A_\alpha \ | \ \alpha \in I\}$ of subsets of *X*, there exists a unique, smallest topology $\tau (\Sigma )$ with $\Sigma \subseteq \tau (\Sigma )$ (Dugundji 1966, Theorem 3.1, p. 65). The family $\tau (\Sigma )$ consists of $\emptyset$, *X*, all finite intersections of the $A_\alpha$, and all arbitrary unions of these finite intersections. $\Sigma$ is called a *subbase* for $\tau (\Sigma )$, and $\tau (\Sigma )$ is said to be *generated* by $\Sigma$. The set of finite intersections of members of $\Sigma$ forms a base for $\tau (\Sigma )$.

### Multi-agent topological model

In this section we define multi-agent models based on topological spaces.

#### Definition 10

Given a topological space $(X, \tau )$, a *neighbourhood function set*$\Phi$ on $(X, \tau )$ is a set of (partial) *neighbourhood functions*$\theta : X\rightharpoonup {\mathcal {A}}\rightarrow \tau$ such that for all $x\in {\mathcal {D}}(\theta )$, for all $i\in {\mathcal {A}}$, and for all $U\in \tau$:

1. $$x\in \theta (x)(i)$$,
2. $$\theta (x)(i)\subseteq {\mathcal {D}}(\theta )$$,
3. for all $y\in X$, if $y\in \theta (x)(i)$ then $y\in {\mathcal {D}}(\theta )$ and $\theta (x)(i)=\theta (y)(i)$,
4. $$\theta |_U\in \Phi $$,

where ${\mathcal {D}}(\theta )$ is the domain of $\theta$, and $\theta |_U$ is the neighbourhood function with ${\mathcal {D}}(\theta |_U)={\mathcal {D}}(\theta )\cap U$ and $\theta |_U(x)(i)= \theta (x)(i)\cap U$.

#### Definition 11

A *multi-agent topological model* (*topo-model*) is a tuple ${\mathcal {M}}=(X, \tau , \Phi , V)$, where $(X, \tau )$ is a topological space, $\Phi$ a neighbourhood function set, and $V: Prop \rightarrow {\mathcal {P}}(X)$ a valuation function. The tuple ${\mathcal X}=(X, \tau , \Phi )$ is a *multi-agent topological frame* (*topo-frame*).

A pair $(x, \theta )$ is called a *neighbourhood situation* if $x\in {\mathcal {D}}(\theta )$. The open set $\theta (x)(i)$ is called an *epistemic neighbourhood at x of agent i*. An epistemic neighbourhood $\theta (x)(i)$ serves as the actual, truthful observation set of the agent *i* at state *x*. This representation is important as we study a notion of knowledge based on observation as in Moss and Parikh (1992). If $(x,\theta )$ is a neighbourhood situation in ${\mathcal {M}}$ we write $(x,\theta )\in {\mathcal {M}}$. Similarly, if $(x,\theta )$ is a neighbourhood situation in ${\mathcal {X}}$ we write $(x,\theta )\in {\mathcal {X}}$.

The following lemma shows that the domain of every neighbourhood function is open.

#### Lemma 12

For any $(X, \tau , \Phi )$ and $\theta \in \Phi$, we have ${\mathcal {D}}(\theta )\in \tau$.

#### Proof

Let $(X, \tau , \Phi )$ be a topo-frame, $\theta \in \Phi$ and $x\in {\mathcal {D}}(\theta )$. By Definition 10, we have $x\in \theta (x)(i)\in \tau$ and $\theta (x)(i)\subseteq {\mathcal {D}}(\theta )$. Therefore, $x\in { Int} ({\mathcal {D}}(\theta ))$. Hence, ${\mathcal {D}}(\theta )={ Int} ({\mathcal {D}}(\theta ))$, i.e., ${\mathcal {D}}(\theta )\in \tau$. $\square$

### Semantics

#### Definition 13

Given a topo-model ${\mathcal {M}}=(X, \tau , \Phi , V)$ and a neighbourhood situation $(x, \theta ) \in {\mathcal {M}}$, the semantics for the language $\mathcal {L}_{{ APAL}_{ int} }$ is defined recursively as:

$$\begin{aligned} \begin{array}{lll} \mathcal {M}, (x, \theta ) \models p &{}\quad \text{ iff }&{}\quad x\in V(p)\\ \mathcal {M}, (x, \theta ) \models \lnot \varphi &{}\quad \text{ iff }&{}\quad \text{ not } \ \mathcal {M}, (x, \theta )\models \varphi \\ \mathcal {M}, (x, \theta )\models \varphi \wedge \psi &{}\quad \text{ iff }&{}\quad \mathcal {M}, (x, \theta )\models \varphi \ \text{ and } \ \mathcal {M}, (x, \theta )\models \psi \\ \mathcal {M}, (x, \theta )\models K_i\varphi &{}\quad \text{ iff }&{}\quad (\forall y \in \theta (x)(i))(\mathcal {M}, (y, \theta )\models \varphi )\\ \mathcal {M}, (x, \theta )\models { int} (\varphi ) &{}\quad \text{ iff }&{}\quad x\in { Int} ( {[\![ \varphi ]\!]^{\theta })}\\ {\mathcal {M}},(x,\theta )\models [\varphi ]\psi &{}\quad \text{ iff } &{}\quad {\mathcal {M}},(x,\theta )\models { int} ( \varphi ) \ \text{ implies } \ {\mathcal {M}},(x,\theta ^\varphi )\models \psi \\ \mathcal {M}, (x, \theta )\models \Box \varphi &{}\quad \text{ iff }&{}\quad (\forall \psi \in {\mathcal {L}}_{{ PAL}_{ int} })(\mathcal {M}, (x, \theta )\models [\psi ]\varphi )\\ \end{array} \end{aligned}$$

where $p\in Prop$, $[\![ \varphi ]\!]^{\theta } =\{ y\in {\mathcal {D}}(\theta ) \ | \ \mathcal {M}, (y, \theta )\models \varphi \}$ and an *updated neighbourhood function*$\theta ^\varphi :X\rightharpoonup {\mathcal {A}}\rightarrow \tau$ is defined such that $\theta ^\varphi =\theta |_{{ Int} [\![ \varphi ]\!]^\theta }$. More precisely, ${\mathcal {D}}(\theta ^\varphi )= { Int} ( [\![ \varphi ]\!]^\theta )$ and $\theta ^\varphi (x)(i)=\theta (x)(i)\cap { Int} ( {[\![ \varphi ]\!]^\theta })$ for all $x\in {\mathcal {D}}(\theta ^\varphi )$.

A formula $\varphi \in \mathcal {L}_{{ APAL}_{ int} }$ is *valid in a topo-model*${\mathcal {M}}$, denoted ${\mathcal {M}}\models \varphi$, iff ${\mathcal {M}}, (x, \theta )\models \varphi$ for all $(x, \theta )\in {\mathcal {M}}$; $\varphi$ is *valid*, denoted $\models \varphi$, iff for all topo-models ${\mathcal {M}}$ we have ${\mathcal {M}}\models \varphi$. Soundness and completeness with respect to topo-models are defined as usual.

Let us now elaborate on the structure of topo-models and the above semantics we have proposed for ${\mathcal {L}}_{{ APAL}_{ int} }$. Given a topo-model $(X, \tau , \Phi , V)$, the epistemic neighbourhoods of each agent at a given state *x* are determined by (partial) functions $\theta : X\rightharpoonup {\mathcal {A}}\rightarrow \tau$ assigning an open neighbourhood to the state in question for each agent. We allow for partial functions in $\Phi$, and close $\Phi$ under restricted functions $\theta |_U$ where $U\in \tau$ (see Definition 10, condition 4) so that updated neighbourhood functions are guaranteed to be well-defined elements of $\Phi$. As in the standard subset space semantics, by picking a neighbourhood situation $(x, \theta )$, we first localize our focus to an *open* subdomain, in fact to ${\mathcal {D}}(\theta )$ (see Lemma 12), including the state *x* and the epistemic neighbourhood of each agent determined by $\theta$ at *x*. The function $\theta (x)$ then designates an epistemic neighbourhood for each agent *i* in ${\mathcal {A}}$. It is guaranteed that every agent *i* is assigned a neighbourhood by $\theta$ at every state *x* in ${\mathcal {D}}(\theta )$, since each $\theta (x)$ is defined to be a *total* function from ${\mathcal {A}}$ to $\tau$. Moreover, condition (1) of Definition 10 ensures that $\emptyset$ cannot be an epistemic neighbourhood, i.e., $\theta (x)(i)\not =\emptyset$ for all $x\in {\mathcal {D}}(\theta )$ and $i\in {\mathcal {A}}$. Finally, conditions (1) and (3) of Definition 10 make sure that the *S*5 axioms for each $K_i$ are sound with respect to all topo-models. We will see in Sect. 5 that our setting allows us to work with the weaker *S*4 notion of knowledge by relaxing the conditions on the neighbourhood functions in $\Phi$.

The semantics proposed for the propositional variables and the Booleans is rather usual both for the standard Kripke semantics and for the classical subset space semantics (Moss and Parikh 1992). In fact, as stated in Proposition 14 below, the truth value of the non-modal formulas depends only on the actual state. While neighbourhood functions, and thus the neighbourhoods defined, play no role in the truth values of these formulas, they are essential in the evaluation of modal formulas, and in capturing observation-based knowledge and information dynamics. We now take a closer look at the semantic clauses for the modalities in $${\mathcal {L}}_{{ APAL}_{ int} }$$ with a particular focus on $$K_i$$ and $${ int} $$.

As also mentioned in Sect. 1, the opens of a topo-model ${\mathcal {M}}=(X, \tau , \Phi , V)$ are considered to be the possible observation sets. In other words, opens of a topology can be considered as the propositions that the agents can in principle observe (but might not have observed yet).^2^ On the other hand, $\theta (x)(i)$ gives us the truthful observation agent *i* currently has at the actual state *x*. Stating the semantic clause for knowledge given in Definition 13 in a slightly different way gives us that

$$\begin{aligned} \mathcal {M}, (x, \theta )\models K_i\varphi \quad \text{ iff }\quad \theta (x)(i)\subseteq [\![ \varphi ]\!]^\theta , \end{aligned}$$

i.e, according to our proposed semantics, agent *i* knows $\varphi$ at *x* (with respect to $\theta$) iff his current truthful observation entails $\varphi$. In particular, this semantic clause implies that the agents cannot know a proposition $\varphi$ unless it is entailed by some possible observation, i.e., by an open set. In this sense, the topology of the model in question restricts the set of propositions the agents can know, based on what they can and cannot observe. We therefore capture an observation-based notion of knowledge in a subset space-like setting by using topological spaces. This obviously goes beyond and enriches the formal treatment of knowledge in terms of the standard relational semantics as the standard relational semantics lacks the ingredients that make it possible to talk about the nature and grounds of acquired knowledge.

The operator $${ int} $$ can be thought of as the most curious modality of the language $${\mathcal {L}}_{{ APAL}_{ int} }$$. Commonly in public announcement logics, it is sufficient for the announcement to be true in order to be announced. But in our logic, following Bjorndahl (2017), the requirement is stronger, capturing an observation-based interpretation of public announcements. More precisely, Bjorndahl (2017) requires not only that the announced formula be true, but also that it be entailed by a piece of truthful observation that the agent could possibly obtain. Given that the elements of $$\tau $$ are taken to be possible observation sets, this can be captured naturally by the topological *interior*. By spelling out the definition of the topological interior operator, we obtain

$$\begin{aligned} \mathcal {M}, (x, \theta )\models { int} (\varphi ) \quad \text{ iff } \quad (\exists U\in \tau )(x\in U \subseteq [\![ \varphi ]\!]^\theta ). \end{aligned}$$

As can be seen in the semantic clause of the public announcement modality, $${ int} $$ behaves as the precondition of the announcement, which constitutes a stronger requirement for announcing $$\varphi $$ than the truth of $$\varphi $$ since $${ Int} ([\![ \varphi ]\!]^\theta ) \subseteq [\![ \varphi ]\!]^\theta $$ (see Bjorndahl 2017, for differences between these two requirements). The precondition $${ int} (\varphi ) $$ therefore requires the *existence* of a truthful observation set entailing the announcement formula $$\varphi $$. In other words, the precondition of an announcement is it being (in principle) *observable*. In this respect, a true proposition cannot be announced if it does not have any *open* subsets including the actual state. For example, on a topo-model with no singleton opens, the agents can never know the actual state (as in Example 1, p. 149 of Georgatos 1994). It is this observation-based interpretation of public announcements that makes Bjorndahl-style topological public announcements different from standard public announcement operators (interpreted via model restrictions). In a framework where knowledge is based on the agent’s current observation set, and every possible observation the agent might acquire later is represented within the given model in terms of open sets of a topology, the operator $${ int} $$ as the precondition for learning something seems to be the right notion to consider. It is a good fit with the intuition behind the subset space/topological semantics. Strengthening the precondition of a formula-parametrized epistemic action is also common in logics of protocols (van Benthem et al. 2009). There is also an obvious, one-way relation between the modalities $$K_i$$ and $${ int} $$. While the semantics of $$K_i\varphi $$ refers to a particular open of the form $$\theta (x)(i)$$ that represents the agent’s current, truthful observation entailing $$\varphi $$, the truth condition for $${ int} (\varphi ) $$ demands only *existence* of such an open (without referring to any particular element of $$\tau $$ or to any agent $$i\in {\mathcal {A}}$$). Therefore, the former claim “having a truthful observation entailing $$\varphi $$” implies the latter existential claim on observation. We thus have $$K_i\varphi \rightarrow { int} (\varphi ) $$ valid with respect to our topological semantics (see also Table 1, axiom ($$K_{ int} $$)), however, the other direction does not always hold: existence of an open *U* with $$x\in U\subseteq [\![ \varphi ]\!]^\theta $$ does not guarantee that $$U=\theta (x)(i)$$.

In general in public announcement logics, the effect of a public announcement is interpreted as model restriction by eliminating the states where the announced formula is not true (van Ditmarsch et al. 2007; Balbiani et al. 2013; van Benthem 2007). Therefore, information gain via public announcements leads to a model change with respect to the aforementioned approach. However, inspired by the intuition behind the subset space semantics and its dynamic modality effort, the information increase in our setting is modelled locally as shrinkage of the initial open neighbourhood to a smaller open neighbourhood without leading to a global change of the model in question.

As usual, the announcement of a formula by an external source in our setting does not depend on the epistemic state of the agents but depends only on whether its precondition is satisfied in the actual state, more specifically in our case, whether it is satisfied by the actual neighbourhood situation. Therefore, given that knowledge of each agent at a neighbourhood situation $$(x, \theta )$$ is evaluated within the open set defined by the function $$\theta $$ at the state *x*, we want the effect of an announcement of $$\varphi $$ to be the shrinkage of $${\mathcal {D}}(\theta )$$ to its largest open subset where $$\varphi $$ is true with respect to the same neighbourhood function. Since the modality $${ int} $$ is evaluated as the topological operator $${ Int} $$, we obtain exactly the desired result as a consequence of the announcement of $$\varphi $$: (1) we preserve the evaluation structure by restricting the initial *open* state space $${\mathcal {D}}(\theta )$$ to an open set again, in particular, to the open set $${ Int} ([\![ \varphi ]\!]^\theta ) $$ induced by the formula $$\varphi $$ with respect to the neighbourhood function $$\theta $$, (2) since the topological interior operator $${ Int} $$ gives the *largest* open set where $$\varphi $$ is true with respect to $$\theta $$, the precondition of an announcement in this setting is not too strong compared to the precondition of being merely true in the sense that the agents can obtain knowledge only via opens sets. To this end, the $${ int} $$ operator enables us to control the shrinkage induced by an announcement in an optimal way.

We now provide some semantic results. As usual in the subset space setting, the truth of non-modal formulas only depends on the state in question:

#### Proposition 14

Given a topo-model ${\mathcal {M}}=(X, \tau , \Phi , V)$, neighbourhood situations $(x, \theta _1), (x, \theta _2)\in {\mathcal {M}}$, and a formula $\varphi \in {\mathcal {L}}_{Pl}$, $(x, \theta _1)\models \varphi \text{ iff } (x, \theta _2)\models \varphi .$

Moreoever, the precondition modality $${ int} $$ corresponds exactly the topological interior operator $${ Int} $$:

#### Proposition 15

Given $${\mathcal {M}}=(X, \tau , \Phi , V)$$, $$\theta \in \Phi $$ and $$\varphi \in {\mathcal {L}}_{{ APAL}_{ int} }$$,

$$\begin{aligned}{}[\![ { int} (\varphi ) ]\!]^\theta ={ Int} ([\![ \varphi ]\!]^\theta ) . \end{aligned}$$

#### Proof

$$\begin{aligned} \begin{array}{llll} [\![ { int} (\varphi ) ]\!]^\theta &{} = &{} \{y\in {\mathcal {D}}(\theta ) \ | \ (y, \theta )\models { int} (\varphi ) \} \\ &{} = &{} \{y\in {\mathcal {D}}(\theta ) \ | \ y\in { Int} ([\![ \varphi ]\!]^\theta ) \} \\ &{} = &{} { Int} ([\![ \varphi ]\!]^\theta ) \ &{} (\text{ since }~{ Int} ([\![ \varphi ]\!]^\theta ) \subseteq {\mathcal {D}}(\theta ))\\ \end{array} \end{aligned}$$

$$\square $$

#### Corollary 16

For any topo-model ${\mathcal {M}}=(X, \tau , \Phi , V)$, $\theta \in \Phi$ and $\varphi \in {\mathcal {L}}_{{ APAL}_{ int} }$,

1. $${ Int} ([\![ { int} (\varphi ) ]\!]^\theta ) ={ Int} ( { Int} ([\![ \varphi ]\!]^\theta ) )={ Int} ([\![ \varphi ]\!]^\theta ) $$, and
2. $$\theta ^\varphi = \theta ^{{ int} (\varphi ) }$$.

#### Proof

Here we only show the second item. By Definition 13 and Proposition 15, we obtain

$$\begin{aligned} {\mathcal {D}}(\theta ^\varphi )={ Int} ( [\![ \varphi ]\!]^\theta ) = { Int} ( [\![ { int} (\varphi ) ]\!]^\theta )={\mathcal {D}}(\theta ^{{ int} (\varphi ) }). \end{aligned}$$

Therefore, both $\theta ^\varphi$ and $\theta ^{{ int} (\varphi ) }$ are defined for the same states. Moreover, for any $x\in {\mathcal {D}}(\theta ^\varphi )$ and any $i\in {\mathcal {A}}$,

$$\begin{aligned} \theta ^\varphi (x)(i)=\theta (x)(i)\cap { Int} ( [\![ \varphi ]\!]^\theta ) = \theta (x)(i)\cap { Int} ( [\![ { int} (\varphi ) ]\!]^\theta )=\theta ^{{ int} (\varphi ) }(x)(i). \end{aligned}$$

Therefore, $$\theta ^\varphi = \theta ^{{ int} (\varphi ) }$$. $$\square $$

#### Proposition 17

1. $$\models [\varphi ]\psi \leftrightarrow [{ int} (\varphi ) ]\psi $$
2. $$\models ({ int} (\varphi ) \wedge \langle \varphi \rangle { int} (\psi ) )\leftrightarrow \langle \varphi \rangle { int} (\psi ) $$

#### Proof

We only show the first item.

$$\begin{aligned} \begin{array}{llll} &{} &{}\quad (x, \theta )\models [\varphi ]\psi \\ &{} \text{ iff } &{}\quad (x,\theta )\models int(\varphi )\text { implies } (x,\theta ^\varphi )\models \psi \\ &{} \text{ iff } &{}\quad (x,\theta )\models { int} ( int(\varphi )) \text { implies } (x,\theta ^\varphi )\models \psi &{}\quad \text{(by } \text{ Corollary } \text{16.1) } \\ &{} \text{ iff } &{}\quad (x,\theta )\models { int} ( int(\varphi )) \text { implies } (x,\theta ^{{ int} (\varphi ) })\models \psi &{}\quad \text{(by } \text{ Corollary } \text{16.2) } \\ &{} \text{ iff } &{}\quad (x,\theta )\models [{ int} (\varphi ) ]\psi \\ \end{array} \end{aligned}$$

$$\square $$

#### Proposition 18

For any topo-model ${\mathcal {M}}=(X, \tau , \Phi , V)$, $\theta \in \Phi$ and $\varphi ,\psi \in {\mathcal {L}}_{{ APAL}_{ int} }$, we have

1. $$[\![ \psi ]\!]^{\theta ^\varphi } = [\![ \langle \varphi \rangle \psi ]\!]^\theta $$, and
2. $$(\theta ^\varphi )^\psi = \theta ^{\langle \varphi \rangle { int} (\psi ) }$$.

#### Proof

Let ${\mathcal {M}}=(X, \tau , \Phi , V)$ be a topo-model, $\theta \in \Phi$ and $\varphi , \psi \in {\mathcal {L}}_{{ APAL}_{ int} }$.

1. $$\begin{aligned} \begin{array}{llll} [\![ \psi ]\!]^{\theta ^\varphi } &{} = &{} \{y\in {\mathcal {D}}(\theta ^\varphi ) \ | \ (y, \theta ^\varphi ) \models \psi \}\\ &{} = &{} \{y\in { Int} ([\![ \varphi ]\!]^\theta ) \ | \ (y, \theta ^\varphi ) \models \psi \} &{}\quad \text{(*) }\\ &{} = &{} \{y\in {\mathcal {D}}(\theta ) \ | \ y\in { Int} ([\![ \varphi ]\!]^\theta ) \ \text{ and } \ (y, \theta ^\varphi ) \models \psi \} &{}\quad \text{(**) }\\ &{} = &{} \{y\in {\mathcal {D}}(\theta ) \ | \ (y, \theta ) \models \langle \varphi \rangle \psi \}\\ &{} = &{} [\![ \langle \varphi \rangle \psi ]\!]^\theta \end{array} \end{aligned}$$
  (*): since ${\mathcal {D}}(\theta ^\varphi ) = { Int} ([\![ \varphi ]\!]^\theta )$ and (**): since ${ Int} ([\![ \varphi ]\!]^\theta ) \subseteq {\mathcal {D}}(\theta )$.
2. By Definition 13, we have that ${\mathcal {D}}(\theta ^{\langle \varphi \rangle { int} (\psi ) })={ Int} ([\![ \langle \varphi \rangle { int} (\psi ) ]\!]^\theta )$, and ${\mathcal {D}}((\theta ^\varphi )^\psi )={ Int} ([\![ \psi ]\!]^{\theta ^\varphi })$. Proposition 18.1 implies $[\![ { int} (\psi ) ]\!]^{\theta ^\varphi }=[\![ \langle \varphi \rangle { int} (\psi ) ]\!]^\theta .$ Then, by Proposition 15, we obtain
  $$\begin{aligned} {\mathcal {D}}((\theta ^\varphi )^\psi )= & {} { Int} ([\![ \psi ]\!]^{\theta ^\varphi }) \\= & {} [\![ { int} (\psi ) ]\!]^{\theta ^\varphi }\\= & {} { Int} ([\![ { int} (\psi ) ]\!]^{\theta ^\varphi }) \\= & {} { Int} ([\![ \langle \varphi \rangle { int} (\psi ) ]\!]^\theta ) \\= & {} {\mathcal {D}}(\theta ^{\langle \varphi \rangle { int} (\psi ) }). \end{aligned}$$
  Therefore, both $(\theta ^\varphi )^\psi$ and $\theta ^{\langle \varphi \rangle { int} (\psi ) }$ are defined for the same states. Moreover, for any $x\in {\mathcal {D}}((\theta ^\varphi )^\psi )$ and $i\in {\mathcal {A}}$, we have
  $$\begin{aligned} \begin{array}{llll} &{} &{} (\theta ^\varphi )^\psi (x)(i)\\ &{} = &{} \theta ^\varphi (x)(i)\cap { Int} ([\![ \psi ]\!]^{\theta ^\varphi }) \\ \ &{} = &{} \theta (x)(i)\cap { Int} ([\![ \varphi ]\!]^\theta ) \cap { Int} ([\![ \psi ]\!]^{\theta ^\varphi }) \\ \ &{} = &{} \theta (x)(i)\cap [\![ { int} (\varphi ) ]\!]^\theta \cap [\![ { int} (\psi ) ]\!]^{\theta ^\varphi } &{}\quad (\text{ by } \text{ Proposition } \text{15 })\\ \ &{} = &{} \theta (x)(i)\cap [\![ { int} (\varphi ) ]\!]^\theta \cap [\![ \langle \varphi \rangle { int} (\psi ) ]\!]^\theta &{}\quad (\text{ by } \text{ Proposition } \text{18.1 })\\ \ &{} = &{} \theta (x)(i)\cap [\![ { int} (\varphi ) \wedge \langle \varphi \rangle { int} (\psi ) ]\!]^\theta \\ \ &{} = &{} \theta (x)(i)\cap [\![ \langle \varphi \rangle { int} (\psi ) ]\!]^\theta &{}\quad (\text{ by } \text{ Proposition } \text{17.2 })\\ \ &{} = &{} \theta (x)(i)\cap { Int} ([\![ \langle \varphi \rangle { int} (\psi ) ]\!]^\theta ) &{}\quad (\text{ since }~[\![ \langle \varphi \rangle { int} (\psi ) ]\!]^\theta \in \tau )\\ \ &{} = &{} \theta ^{\langle \varphi \rangle { int} (\psi ) }(x)(i) \end{array} \end{aligned}$$
  where $[\![ \langle \varphi \rangle { int} (\psi ) ]\!]^\theta \in \tau$ follows from Proposition 15 and Proposition 18.1 by
  $$\begin{aligned} { Int} [\![ \psi ]\!]^{\theta ^\varphi } =[\![ { int} (\psi ) ]\!]^{\theta ^\varphi }=[\![ \langle \varphi \rangle { int} (\psi ) ]\!]^\theta . \end{aligned}$$
  Therefore, we conclude that $(\theta ^\varphi )^\psi = \theta ^{\langle \varphi \rangle { int} (\psi ) }$.

$$\square $$

### The least topological model

Recalling the semantics for ${\mathcal {L}}_{{ APAL}_{ int} }$ proposed in Sect. 2.4, given a topo-model ${\mathcal {M}}=(X, \tau , \Phi , V)$ every formula is evaluated with respect to a pair called neighbourhood situation $(x, \theta )$ in ${\mathcal {M}}$ such that $x\in {\mathcal {D}}(\theta )\subseteq X$. Since the neighbourhood functions may be partial and the epistemic neighbourhoods are defined via these functions, we do not necessarily use the whole domain of the topo-model in question in the evaluation of the formulas but only the states for which a neighbourhood function is defined. In other words, for any topo-model ${\mathcal {M}}=(X, \tau , \Phi , V)$, only the states in

$$\begin{aligned} {\mathcal {D}}(\Phi ):{=} \bigcup \{{\mathcal {D}}(\theta ) \ | \ \theta \in \Phi \} \end{aligned}$$

are concerned with the truth value of the formulas in ${\mathcal {L}}_{{ APAL}_{ int} }$. In this section we describe topo-models that are indistinguishable on that domain restriction. Roughly speaking, we will categorize the topo-models with respect to their neighbourhood function sets and show that the class of all topo-models having the “equivalent” neighbourhood function sets can be partitioned in such a way that the elements of the same equivalence class are modally equivalent with respect to ${\mathcal {L}}_{{ APAL}_{ int} }$ and each equivalence class has a *minimal* element.

In order to be able define such a partition, we first need to make precise what we mean by partial functions, and correspondingly, neighbourhood function sets being equivalent. Given any two partial functions $\theta : X\rightharpoonup Y$ and $\theta ':X'\rightharpoonup Y'$, we say $\theta$ and $\theta '$ are *equivalent*, denoted by $\theta \equiv \theta '$ iff

1. $${\mathcal {D}}(\theta )={\mathcal {D}}(\theta ')$$, and
2. for all $$x\in {\mathcal {D}}(\theta )$$, $$\theta (x)=\theta '(x)$$.

Informally speaking, two partial functions are equivalent if and only if they give the same *total* function when they are restricted to their respective domains. In particular, for any two equivalent partial functions $\theta : X\rightharpoonup Y$ and $\theta ':X'\rightharpoonup Y'$, it might be the case that $X\not =X'$. Similarly, we say two neighbourhood function sets $\Phi$ and $\Phi '$ (defined on $(X, \tau )$ and $(X', \tau ')$, respectively) are *equivalent*, denoted by $\Phi \equiv \Phi '$, iff there is a bijection $f:\Phi \rightarrow \Phi '$ such that $\theta \equiv f(\theta )$ for all $\theta \in \Phi$.

Let ${\mathfrak {F}}$ (${\mathfrak {K}}$) denote the class of all topo-frames (topo-models) and ${\mathfrak {F}}_\Phi$ (${\mathfrak {K}}_\Phi$) denote the class of all topo-frames (topo-models) whose neighbourhood function set is equivalent to $\Phi$. Intuitively speaking, even if the topo-frames in ${\mathfrak {F}}_\Phi$ can be based on different topological spaces (both the spaces and the topologies may vary), ${\mathfrak {F}}_\Phi$ groups together the topo-frames whose neighbourhood functions behave exactly the same way, in particular, as the ones in $\Phi$. We therefore slightly abuse the notation and denote the neighbourhood function set of each topo-frame (topo-model) in ${\mathfrak {F}}_\Phi$ (${\mathfrak {K}}_\Phi$) by $\Phi$. Essentially, every topo-frame in ${\mathfrak {F}}_\Phi$ has the same set of neighbourhood situations (modulo the above defined equivalence) and we write $(x, \theta )\in {\mathfrak {F}}_\Phi$ if $(x, \theta )$ is a neighbourhood situation of a topo-frame in ${\mathfrak {F}}_\Phi$ (and similarly for ${\mathfrak {K}}_\Phi$). Moreover, for all ${\mathcal {X}}=(X, \tau , \Phi )\in {\mathfrak {F}}_\Phi$, we have (1) ${\mathcal {D}}(\Phi )\subseteq X$ and (2) $\{\theta (x)(i) \ | \ (x, \theta )\in {\mathcal {X}}, i\in {\mathcal {A}}\}\subseteq \tau$ (otherwise $\Phi$ could not be defined on $(X, \tau )$, see Definition 11). Lastly, given a topo-model ${\mathcal {M}}=(X, \tau , \Phi , V)\in {\mathfrak {K}}_\Phi$, we define

$$\begin{aligned} {\mathfrak {K}}_{\Phi , V} :{=}\{{\mathcal {M}}'\in {\mathfrak {K}}_\Phi \ | \ {\mathcal {M}}'=(X', \tau ', \Phi , V') \quad \text{ and }\quad V'|_{{\mathcal {D}}(\Phi )} = V|_{{\mathcal {D}}(\Phi )}\}, \end{aligned}$$

i.e., ${\mathfrak {K}}_{\Phi , V}$ is the class of all topo-models carrying the neighbourhood function set $\Phi$ and whose valuation functions coincide with *V* on ${\mathcal {D}}(\Phi )$. Observe that the set of all ${\mathfrak {K}}_{\Phi , V}\subseteq {\mathfrak {K}}_\Phi$ partitions the class ${\mathfrak {K}}_\Phi$.

For any frames ${\mathcal {X}}_1=(X_1, \tau _1, \Phi ), {\mathcal {X}}_2=(X_2, \tau _2, \Phi ) \in {\mathfrak {F}}_\Phi$,

$$\begin{aligned} {\mathcal {X}}_1\sqsubseteq _\Phi {\mathcal {X}}_2\quad \text{ iff }\quad X_1\subseteq X_2 \quad \text{ and }\quad \tau _1\subseteq \tau _2. \end{aligned}$$

Clearly, $\sqsubseteq _\Phi$ is also a partial order. We say ${\mathcal {M}}=(X, \tau , \Phi , V)$ is *a minimal model* in ${\mathfrak {K}}_{\Phi , V}$ if its frame is a minimal frame in ${\mathfrak {F}}_\Phi$ with respect to $\sqsubseteq _\Phi$.

#### Proposition 19

1. There exists a unique minimal frame (the least frame) in each ${\mathfrak {F}}_\Phi$.
2. There exist a unique minimal model (the least model) in each ${\mathfrak {K}}_{\Phi , V}$.

#### Proof

1. Let ${\mathcal {X}}=(X, \tau , \Phi )$ be a topo-frame in ${\mathfrak {F}}$. Consider the topology $\tau _\Phi$ generated by $\Sigma _\Phi :{=}\{\theta (x)(i) \ | \ (x, \theta )\in {\mathfrak {F}}_\Phi , i\in {\mathcal {A}}\}$, i.e., by the set of all epistemic neighbourhoods defined by the neighbourhood functions in $\Phi$. As $\Sigma _\Phi$ covers ${\mathcal {D}}(\Phi )$, the generated topology $\tau _\Phi$ constitutes the smallest topology on the domain ${\mathcal {D}}(\Phi )$ satisfying conditions (1) ${\mathcal {D}}(\Phi )\subseteq X$ and (2) $\{\theta (x)(i) \ | \ (x, \theta )\in {\mathcal {X}}, i\in {\mathcal {A}}\}\subseteq \tau _\Phi$. Therefore, for all ${\mathcal {X}}=(X, \tau , \Phi )\in {\mathfrak {F}}_\Phi$, ${\mathcal {D}}(\Phi )\subseteq X$ and $\tau _\Phi \subseteq \tau$, i.e., $({\mathcal {D}}(\Phi ), \tau _\Phi , \Phi )\sqsubseteq _\Phi {\mathcal {X}}$.
2. By definition of ${\mathfrak {K}}_{\Phi , V}$, the valuation function of each topo-model in this class coincides on ${\mathcal {D}}(\Phi )$, therefore, the least model in ${\mathfrak {K}}_{\Phi , V}$ is $({\mathcal {D}}(\Phi ), \tau _\Phi , \Phi , V|_{{\mathcal {D}}(\Phi )})$.

$$\square $$

#### Theorem 20

For each class ${\mathfrak {K}}_{\Phi , V}$, all topo-models in ${\mathfrak {K}}_{\Phi , V}$ are modally equivalent with respect to ${\mathcal {L}}_{{ APAL}_{ int} }$.

#### Proof

We show that for any ${\mathcal {M}}_1=(X_1, \tau _1, \Phi , V_1), {\mathcal {M}}_2=(X_2, \tau _2, \Phi , V_2) \in {\mathfrak {K}}_{\Phi , V}$, $(x, \theta )\in {\mathfrak {K}}_\Phi$, and $\varphi \in {\mathcal {L}}_{{ APAL}_{ int} }$:

$$\begin{aligned} {\mathcal {M}}_1, (x, \theta )\models \varphi \quad \text{ iff } \quad {\mathcal {M}}_2, (x, \theta )\models \varphi . \end{aligned}$$

The proof follows by $$<^S_d $$ induction on $$\varphi $$, where the case $$\varphi = [\psi ]\chi $$ is proved by a subinduction on $$\chi $$. Here we only show the base case $$\varphi =p$$, the case for $$\varphi ={ int} (\psi ) $$, and the subinductive clauses $$\chi =p$$ and $$\chi =\Box \sigma $$ for case announcement $$\varphi =[\psi ]\chi $$. The inductive cases negation, conjunction and $$K_i$$ follow from Lemma 6.2, and the subinduction on $$\chi $$ for case announcement $$\varphi =[\psi ]\chi $$ follows from Lemma 7, and finally the case for $$\varphi =\Box \psi $$ follows from Lemma 6.5.

**Base Case:**$$\varphi :{=} p$$

$$\begin{aligned} \begin{array}{llll} {\mathcal {M}}_1, (x, \theta )\models p &{}\quad \text{ iff } &{}\quad x\in V_1(p) &{} \ \\ &{}\quad \text{ iff } &{}\quad x\in V_1(p)\cap {\mathcal {D}}(\Phi )&{} \text{ since }~x\in {\mathcal {D}}(\theta ) \\ &{}\quad \text{ iff } &{}\quad x\in V_2(p)\cap {\mathcal {D}}(\Phi )&{} \text{ since }~V_1(p)\cap {\mathcal {D}}(\Phi )=V_2(p)\cap {\mathcal {D}}(\Phi )\\ &{}\quad \text{ iff } &{}\quad x\in V_2(p) &{} \text{ since }~x\in {\mathcal {D}}(\theta ) \\ &{}\quad \text{ iff } &{}\quad {\mathcal {M}}_2, (x, \theta )\models p &{} \end{array} \end{aligned}$$

**Inductive Hypothesis (IH)**: For all formulas $\psi \in {\mathcal {L}}_{{ APAL}_{ int} }$, if $\psi <^S_d \varphi$, then ${\mathcal {M}}_1, (x, \theta )\models \psi \ \text{ iff } \ {\mathcal {M}}_2, (x, \theta )\models \psi$, for any $(x, \theta )\in {\mathfrak {K}}_\Phi$.

**Case**$$\varphi :{=} { int} (\psi ) $$.

Let ${ Int} _ 1$ and ${ Int} _ 2$ denote the interior operators of the topological spaces $(X_1, \tau _1)$ and $(X_2, \tau _2)$, respectively.

- ($\Rightarrow$) Suppose ${\mathcal {M}}_1, (x, \theta )\models { int} (\psi )$, i.e., $x\in { Int} _ 1\{y\in {\mathcal {D}}(\theta ) \ | \ {\mathcal {M}}_1, (y, \theta )\models \psi \}$. By Lemma 6.2 and (IH) we have
  $$\begin{aligned} \{y\in {\mathcal {D}}(\theta ) \ | \ {\mathcal {M}}_1, (y, \theta )\models \psi \} = \{y\in {\mathcal {D}}(\theta ) \ | \ {\mathcal {M}}_2, (y, \theta )\models \psi \}, \end{aligned}$$
  thus, $x\in { Int} _ 1\{y\in {\mathcal {D}}(\theta ) \ | \ {\mathcal {M}}_2, (y, \theta )\models \psi \}$. Therefore, there is an open $U\in \tau _1$ such that
  $$\begin{aligned} x\in U\subseteq \{y\in {\mathcal {D}}(\theta ) \ | \ {\mathcal {M}}_2, (y, \theta )\models \psi \}. \end{aligned}$$
  Now consider the set ${\mathcal {D}}(\theta )\cap U$. It is non-empty since $x\in {\mathcal {D}}(\theta )\cap U$. Moreover, ${\mathcal {D}}(\theta )\cap U ={\mathcal {D}}(\theta |_U)$ and $\theta |_U\in \Phi$. Therefore, ${\mathcal {D}}(\theta |_U)\in \tau _2$ since ${\mathcal {M}}_2\in {\mathfrak {K}}_\Phi$. And, obviously, $x\in {\mathcal {D}}(\theta )\cap U={\mathcal {D}}(\theta |_U)\subseteq \{y\in {\mathcal {D}}(\theta ) \ | \ {\mathcal {M}}_2, (y, \theta )\models \psi \}$, hence, $x\in { Int} _ 2 \{y\in {\mathcal {D}}(\theta ) \ | \ {\mathcal {M}}_2, (y, \theta )\models \psi \}$ meaning that ${\mathcal {M}}_2, (x, \theta )\models { int} (\psi )$.
- ($\Leftarrow$) Similar to the above case. **Case**$\varphi :{=} [\psi ]\chi$. This case follows from a subinduction on $\chi$. Here we only show the case $\chi =p$ and $\chi =\Box \sigma$, and the other cases are equally elementary and follow from Lemma 7 and the validities (R2)–(R6) appear in the axiomatization in Table 1. **Subcase**$\varphi :{=} [\psi ]p$
  $$\begin{aligned} \begin{array}{llll} {\mathcal {M}}_1, (x, \theta )\models [\psi ]p &{}\quad \text{ iff } &{}\quad {\mathcal {M}}_1, (x, \theta )\models { int} (\psi ) \rightarrow p &{}\quad \text{ by } \text{ the } \text{ validity } \text{(R1) } \\ &{}\quad \text{ iff } &{}\quad {\mathcal {M}}_2, (x, \theta )\models { int} (\psi ) \rightarrow p &{}\quad \text{ by } \text{ Lemma } \text{7.1 } \text{ and } \text{(IH) }\\ &{}\quad \text{ iff } &{}\quad {\mathcal {M}}_2, (x, \theta )\models [\psi ]p &{}\quad \text{ by } \text{ the } \text{ validity } \text{(R1) } \\ \end{array} \end{aligned}$$
  **Subcase**$\varphi :{=} [\psi ] \Box \sigma$ For all $\eta \in {\mathcal {L}}_{{ PAL}_{ int} }$, $[\psi ][\eta ]\sigma <^S_d [\psi ] \Box \sigma$, as $[\psi ] \Box \sigma$ has one more $\Box$ than $[\psi ][\eta ]\sigma$.
  $$\begin{aligned} \begin{array}{ll} &{}\quad {\mathcal {M}}_1, (x, \theta )\models [\psi ] \Box \sigma \\ \text{ iff } &{}\quad {\mathcal {M}}_1, (x, \theta )\models { int} (\psi ) \ \text{ implies } \ {\mathcal {M}}_1, (x, \theta ^\psi )\models \Box \sigma \\ \text{ iff } &{}\quad {\mathcal {M}}_1, (x, \theta )\models { int} (\psi ) \ \text{ implies } \ \ (\forall \eta \in {\mathcal {L}}_{{ PAL}_{ int} })({\mathcal {M}}_1, (x, \theta ^\psi )\models [\eta ]\sigma ) \\ \text{ iff } &{}\quad (\forall \eta \in {\mathcal {L}}_{{ PAL}_{ int} })({\mathcal {M}}_1, (x, \theta )\models { int} (\psi ) \ \text{ implies } \ {\mathcal {M}}_1, ( x, \theta ^\psi )\models [\eta ]\sigma ) \\ \text{ iff } &{}\quad (\forall \eta \in {\mathcal {L}}_{{ PAL}_{ int} }) ({\mathcal {M}}_1, (x, \theta )\models [\psi ][\eta ]\sigma ) \\ \text{ iff } &{}\quad (\forall \eta \in {\mathcal {L}}_{{ PAL}_{ int} }) ({\mathcal {M}}_2, (x, \theta )\models [\psi ][\eta ]\sigma )\quad \text{* }\\ \text{ iff } &{}\quad {\mathcal {M}}_2, (x, \theta )\models [\psi ] \Box \sigma \qquad \text{(by } \text{ a } \text{ similar } \text{ argument) } \\ \end{array} \end{aligned}$$
  (*): by $[\psi ][\eta ]\sigma <^S_d [\psi ] \Box \sigma$ and (IH)

$$\square $$

#### Corollary 21

Each class ${\mathfrak {K}}_{\Phi , V}$ can be represented by its least element $({\mathcal {D}}(\Phi ), \tau _\Phi , \Phi , V|_{{\mathcal {D}}(\Phi )})$ up to modal equivalence.

### Examples

In this section we present two examples demonstrating how our multi-agent topological semantics works. The first example is a multi-agent version of an example presented in Bjorndahl (2017) for Bjorndahl’s single-agent setting and the second one is concerned with two agents learning bit by bit (finite) prefixes of a pair of infinite binary sequences.

#### The Jewel in the Tomb

**Fig. 1:**
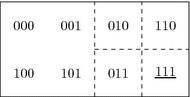
Dashed squares represent the elements of the basis generating the topology $\tau$

We illustrate our semantics by means of a multi-agent version of Bjorndahl’s example in Bjorndahl (2017) about the jewel in the tomb. Indiana Jones (*i*) and Emile Belloq (*e*) are both scouring for a priceless jewel placed in a tomb. The tomb could either contain a jewel or not, the tomb could have been rediscovered in modern times or not, and (beyond Bjorndahl 2017), the tomb could be in the Valley of Tombs in Egypt or not. The propositional variables corresponding to these propositions are, respectively, *j*, *d*, and *t*. We represent a valuation of these variables by a triple *xyz*, where $x,y,z \in \{0,1\}$. Given the carrier set $X = \{ xyz \mid x,y,z \in \{0,1\} \}$, the topology $\tau$ that we consider is generated by the basis consisting of the subsets $\{000,100,001,101\}$, $\{010\}$, $\{110\}$, $\{011\}$, $\{111\}$ (see Fig. 1). The idea is that one can only conceivably know (or learn) about the jewel or the location on condition that the tomb has been discovered. Therefore, $\{000,100,001,101\}$ has no strict subsets besides the empty set: if the tomb has not yet been discovered, no one can have any information about the jewel or the location. However, provided that the tomb has been discovered, the agents might know whether or not it contains a jewel, and/or whether it is the Valley of Tombs in Egypt. In this example, we stipulate that the actual state is 111.

**Fig. 2:**
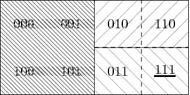
Patterned sets represent Emile’s neighbourhoods defined by $\theta '$: $\theta '(111)(e) =\theta '(011)(e) = \{ {111}, {011} \}$, $\theta '(010)(e) =\theta '(110)(e) = \{ {010}, {110} \}$, $\theta '(000)(e) =\theta '(100)(e) =\theta '(001)(e)=\theta '(101)(e) = \{ {000}, {100}, {001}, {101} \}$

A topo-model ${\mathcal {M}}=(X, \tau , \Phi , V)$ for this topology $(X,\tau )$ has $\Phi$ as the set of all neighbourhood functions that are partitions of *X* for both agents, and restrictions of these functions to open sets. A typical $\theta \in \Phi$ describes complete ignorance of both agents and is defined as $\theta (w)(i) = \theta (w)(e) = X$ for all $w\in X$. A more interesting neighbourhood situation in this model is one wherein Indiana and Emile have different knowledge. Let us assume that Emile has the advantage over Indiana so far, as he knows the location of the tomb but Indiana does not. This is the $\theta '$ such that for all $w \in X$, $\theta '(w)(i) = X$, whereas the partition for Emile consists of sets $\{000,100,001,101\}$, $\{{110},{010}\}$, $\{{111},{011}\}$, i.e., $\theta '(111)(e)=\{{111},{011}\}$, etc (see Fig. 2).

We now can evaluate what Emile knows about Indiana at 111. Firstly, Emile knows that the tomb is in the Valley of Tombs in Egypt

1 $$\begin{aligned} {\mathcal {M}}, ({111}, \theta ') \models K_e t \end{aligned}$$

and he also knows that Indiana does not know that:

2 $$\begin{aligned} {\mathcal {M}}, ({111}, \theta ') \models K_e \lnot (K_i \lnot t \vee K_i t) \end{aligned}$$

The statement (2) involves verifying ${\mathcal {M}}, ({w}, \theta ') \models \hat{K}_i t$ and ${\mathcal {M}}, ({w}, \theta ') \models \hat{K}_i \lnot t$ for all $w\in \theta '(111)(e)=\{ {111}, {011}\}$, which is Emile’s current epistemic range. And this is true for both elements 111 and 110 of $\theta '(111)(e)$, because $\theta '(110)(i)=\theta '(111)(i) = X$, and $000, 001 \in X$, and while ${\mathcal {M}}, (001, \theta ') \models t$, we also have ${\mathcal {M}}, (000, \theta ') \models \lnot t$. We can also check that Emile knows that Indiana considers it possible that Emile doesn’t know the tomb’s location:

3 $$\begin{aligned} {\mathcal {M}}, ({111}, \theta ') \models K_e \hat{K}_i \lnot (K_e t \vee K_e \lnot t) \end{aligned}$$

Evaluating this goes beyond Emile’s initial epistemic range $\{111, 011\}$ because, e.g., for $111\in \theta '(111)(e)$, we have ${\mathcal {M}}, ({111}, \theta ') \models \hat{K}_i \lnot (K_e t \vee K_e \lnot t)$ iff there exists $y_0\in \theta '(111)(i)$ such that $(y_0, \theta ')\models \lnot K_e t\wedge \lnot \hat{K}_e\lnot t$. Therefore, such an element $y_0$ cannot be in Emile’s initial epistemic range $\{111, 011\}$, since $(111, \theta ')\models K_e t$ and $(011, \theta )\models K_et$. In fact, it has to be the case that $y_0\in \{000, 001, 100, 101\}$. This situation however does not create any problems in our setting since $(y_0, \theta ')$ is a well-defined neighbourhood situation, and Emile’s epistemic range at $y_0$ is defined by $\theta '$ as $\theta '(y_0)(e)= \{000, 001, 100, 101\}$.

Given their prior knowledge, announcements will change Emile and Indiana’s knowledge in different ways. Consider the announcement of *j*. An important point to notice is that the announcement of *j* does not only convey the information $[\![ j]\!]^{\theta '}=\{100, 101, 110, 111\}$ but that it also leads to learning ${ Int} ([\![ j]\!]^{\theta '}) =\{110, 111\}$. This corresponds exactly to the fact that one can know about the jewel on the condition that the tomb has already been rediscovered. Therefore, the announcement of *j* evidences the fact that the tomb has already been discovered, hence, it conveys more information than only *j* being true. This results in Emile knowing everything but Indiana still being uncertain about the location:

4 $$\begin{aligned} {\mathcal {M}}, ({111}, \theta ') \models [j] (K_e (j\wedge d\wedge t) \wedge K_i (j\wedge d) \wedge \lnot (K_i t \vee K_i \lnot t)) \end{aligned}$$

**Fig. 3:**
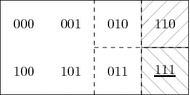
As ${\mathcal {D}}((\theta ')^j)={ Int} ([\![ j]\!]^{\theta '}) =\{111, 110\}$, the updated neighbourhood function $(\theta ')^j$ is defined only for these points. Patterned sets again represent Emile’s neighbourhoods defined by $(\theta ')^j$: $(\theta ')^j(111)(e) = \{ {111}\}$ and $(\theta ')^j(110)(e) = \{ {110} \}$. For Indiana, we have $(\theta ')^j(111)(j)=(\theta ')^j(110)(i)=\{{111}, {110}\}$

Model checking this involves computing the epistemic ranges of both agents given by the updated neighbourhood function $(\theta ')^j$ at 111 (see Fig. 3). Note that ${ Int} ( [\![ j]\!]^{\theta '})=\{111, 110\}$. Therefore, $(\theta ')^j(111)(e)={ Int} ( [\![ j]\!]^{\theta '})\cap \theta '(111)(e) = \{111\}$, and for Indiana $(\theta ')^j(111)(i)={ Int} ( [\![ j]\!]^{\theta '})\cap \theta '(111)(i)=\{111, 110\}$.

There is an announcement after which Emile and Indiana know everything (for example the announcement of $j\wedge t$):

$$\begin{aligned} {\mathcal {M}}, ({111}, \theta ) \models \Diamond (K_e (j \wedge d \wedge t) \wedge K_i (j \wedge d \wedge t)). \end{aligned}$$

Observe that ${ Int} ([\![ j\wedge t]\!]^{\theta '}) =\{111\}$, thus, $(\theta ')^j(111)(e) = (\theta ')^j(111)(j)=\{111\}.$ Again, the announcement of $j\wedge t$ carries the implication that the tomb has been rediscovered. On the other hand, as long as the tomb has not been discovered, nothing will make Emile (or Indiana) learn that it contains a jewel or where the tomb is located:

$$\begin{aligned} {\mathcal {M}}\models \lnot d \rightarrow \Box (\lnot (K_e j \vee K_e \lnot j) \wedge \lnot (K_e t \vee K_e \lnot t)). \end{aligned}$$

#### Binary strings

We begin the example by defining a topology over the set of ordered pairs of binary strings, i.e., the domain of our topology is $X=\{0,1\}^\infty \times \{0,1\}^\infty$.

Note that we can consider *X* to be points in the unit square $[0,1]\times [0,1]$, by looking at each element of $\{0,1\}^\infty$ as the binary representation of a real number in [0, 1]. So for example, $(01000\ldots ,11000\ldots )$ represents (.25, .75). This correspondence is not one-to-one, however, because many points in [0, 1] have more than one possible representation as binary strings. For example, $1000\ldots$ and $0111\ldots$ both represent 0.5. In fact, every fraction of the form $\frac{i}{2^k}$ for some $i,k\in \mathbb N$ with $0<i<2^k$ has two possible representations, while every other element of [0, 1] has a unique representation. Therefore, every element of $[0,1]\times [0,1]$ has either one, two, or four possible representations in $\{0,1\}^\infty \times \{0,1\}^\infty$. So, we can consider each element of $\{0,1\}^\infty \times \{0,1\}^\infty$ to represent one element of $[0,1]\times [0,1]$, but every element of $[0,1]\times [0,1]$ does not represent a unique element of $\{0,1\}^\infty \times \{0,1\}^\infty$.

Let us now introduce some notation. If $s\in \{0,1\}^\infty$, for $n\in \mathbb N^+$, we let $s|_n$ be the first *n* bits of *s*, and we let *s*[*n*] be the *n*th bit of *s*. As usual, we let $\{0,1\}^*$ be the set of finite strings over $\{0,1\}$ and for $d\in \{0,1\}^*$, |*d*| is the length of *d*. For $d\in \{0,1\}^*$ we define $S_d=\{x\in \{0,1\}^\infty \;|\;x|_{|d|}=d\}$, in other words, $S_d$ is the set of all infinite binary strings that have *d* as a prefix. Note that $S_\epsilon$ is $\{0,1\}^\infty$, since $\epsilon$ is the empty string. Note also that when we consider the elements of $\{0,1\}^\infty$ as points on the unit interval, we can think of $S_d$ as a certain subinterval of the unit interval. More precisely, each $S_d$ is the interval bounded by $\frac{d}{2^{|d|}}$ and $\frac{d+1}{2^{|d|}}$ when *d* is viewed as the binary representation of a natural number. As above, we cannot, however, go in the opposite direction and consider all such intervals to be sets of the form $S_d$, since there are multiple possible representations of some of the points in [0, 1] as binary strings.

Now consider the topology $\tau$ generated by the set

$$\begin{aligned} {\mathcal {B}}=\{S_d\;|\;d\in \{0,1\}^*\}. \end{aligned}$$

It is not hard to see that ${\mathcal {B}}$ indeed constitutes a base over the domain $\{0,1\}^\infty$:

1. Since $S_\epsilon \in {\mathcal {B}}$, we have $\bigcup {\mathcal {B}}=\{0,1\}^\infty$.
2. For any $U_1, U_2\in {\mathcal {B}}$, we have either $U_1\cap U_2=\emptyset$, $U_1\cap U_2=U_1$ or $U_1\cap U_2=U_2$. Therefore, ${\mathcal {B}}$ is closed under finite intersections.

For our example, we use the product space $(\{0,1\}^\infty \times \{0,1\}^\infty , \tau \times \tau )$ and we have two agents *a* and *b*. Intuitively speaking, agent *a* is concerned with the bits of the first coordinate and agent *b* is concerned with the bits of the second coordinate encoded as infinite binary strings. Let $\theta _\epsilon ((x,y))(a)=\theta _\epsilon ((x,y))(b)=\{0,1\}^\infty \times \{0,1\}^\infty$, and for $i\in \mathbb N^+$, let $\theta _i((x,y))(a)=S_{x|_i}\times \{0,1\}^\infty$, and let $\theta _i((x,y))(b)=\{0,1\}^\infty \times S_{y|_i}$, where ${\mathcal {D}}(\theta _i)=\{0,1\}^\infty \times \{0,1\}^\infty$. In other words, for agent *a*, $\theta _i$ gives the set of pairs where the first component of the pair agrees with *x* in the first *i* bits, with any possible second value for the pair. Similarly for agent *b*. We note that $\theta _{i+1}$ always is more informative than $\theta _i$. Finally, in order to obtain our neighbourhood function set $\Phi$, we must close the set of functions described above under open domain restriction, so we let $\Phi =\{\theta :X\rightharpoonup \{a, b\}\rightarrow \tau \;|\;\exists i\in \mathbb N^+ \cup \{\epsilon \}, U\in \tau \text { such that }\theta =\theta _i|U\}$. It is easy to see that $\Phi$ satisfies the properties of a neighbourhood function set given in Definition 10.

In order to evaluate formulas on this topo-frame, we define atomic propositions

$$\begin{aligned} { Prop}=\{\mathbf {x}_i \mid i\in \mathbb N^+\} \cup \{\mathbf {y}_i \mid i\in \mathbb N^+\} \end{aligned}$$

where

$$\begin{aligned} V(\mathbf {x}_i)= & {} \{(x,y)\in \{0,1\}^\infty \times \{0,1\}^\infty \mid x[i]=1\}; \\ V(\mathbf {y}_i)= & {} \{(x,y)\in \{0,1\}^\infty \times \{0,1\}^\infty \mid y[i]=1\}. \end{aligned}$$

Intuitively speaking, the propositional variables refer to the *x*- and *y*-coordinates of the pairs of infinite binary strings. We read $\mathbf {x}_i$ as “*theith bit of thex-coordinate is 1*” and $\mathbf {y}_i$ as “*theith bit of they-coordinate is 1*”.

We can now evaluate some formulas on the topo-model

$$\begin{aligned} {\mathcal {M}}=(\{0,1\}^\infty \times \{0,1\}^\infty , \tau \times \tau , \Phi , V) \end{aligned}$$

at the state $(x, y)=(010000\ldots , 110110\ldots )$ and given the initial situation described by the function $\theta _1$. In other words, we have that *a* knows that the first bit of *x* is 0, *b* knows that the first bit of *y* is 1, and both are ignorant about the other’s bits, and this is common knowledge. In formulas, we have

$$\begin{aligned} \begin{array}{ll} {\mathcal {M}}, ((x,y), \theta _1) \models K_a \lnot \mathbf {x}_1 &{}\quad a\,\text {knows that}\,x[1]=0 \\ {\mathcal {M}}, ((x,y), \theta _1) \models K_b \mathbf {y}_1 &{}\quad b\,\text {knows that}~y[1]=1 \\ {\mathcal {M}}, ((x,y), \theta _1) \models K_{a} \lnot (K_{b}\mathbf {x}_1 \vee K_{b}\lnot \mathbf {x}_1) &{}\quad a~\text {knows that}~b \\ &{}~~~\quad \text {does not know the value of}~x[1] \\ {\mathcal {M}}, ((x,y), \theta _1) \models K_{b} \lnot (K_{a}\mathbf {y}_1 \vee K_{a}\lnot \mathbf {y}_1) &{}\quad b~\text {knows that}~a \\ &{}~~~\quad \text {does not know the value of}~y[1]\\ \ldots \text {etc., etc.}&{} \\ \end{array} \end{aligned}$$

Now consider announcements of the following form: given $((x,y),\theta _n)$ (wherein *a* and *b* know up to the *n*th bit of *x* and *y*, respectively), the announcement $\varphi ^{n+1}_x$ is of the form ‘if the *n*th bit of *x* is 1, then the $(n+1)$st bit is *j*, and if the *n*th bit of *x* is 0, then $(n+1)$st bit of *x* is $1-j$’ with the restriction that the announcement is indeed truthful and where $j\in \{0, 1\}$. So it can only be announced for $j=0$ or $j=1$ but not for both. In other words, $\varphi ^{n+1}_x$ is either of the form ‘the *n*th bit of *x* is equal to its $n+1$st bit’ or of the form ‘the *n*th bit of *x* is different from its $(n+1)$st bit’ but they cannot be announced at the same time as only one of them can be truthful. Then this announcement informs *abut not b* of the value of the $(n+1)$st digit of *x*.

**Fig. 4:**
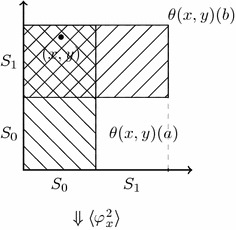
Initial situation where *a* knows the 1st bit of *x* is 0 and *b* knows the first bit of *y* is 1, and both are ignorant about the other’s bit. We have $\theta ((x, y))(a)=S_0\times \{0, 1\}^\infty$ and $\theta ((x, y))(b)=\{0, 1\}^\infty \times S_1$

**Fig. 5:**
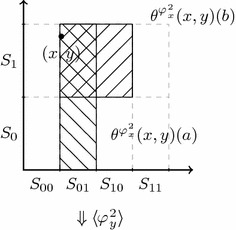
After the announcement of $\varphi ^2_x$, we obtain the following smaller neighbourhoods given by the updated function $\theta ^{\varphi ^2_x}$: $\theta ^{\varphi ^2_x}(x, y)(a)=S_{01}\times \{0, 1\}^\infty$, and $\theta ^{\varphi ^2_x}(x, y)(b)=(S_{01}\cup S_{10})\times S_1$

**Fig. 6:**
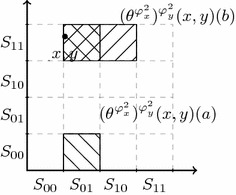
After further announcing $\varphi ^2_y$, the updated function $(\theta ^{\varphi ^2_x})^{\varphi ^2_y}$ gives the neighbourhoods: $(\theta ^{\varphi ^2_x})^{\varphi ^2_y}(x, y)(a)=S_{01}\times (S_{00}\cup S_{11})$, and $(\theta ^{\varphi ^2_x})^{\varphi ^2_y}(x, y)(b)=(S_{01}\cup S_{10})\times S_{11}$

For *b* it is merely an extension of the initial sequences (that he is unable to distinguish anyway, as we will see) with either 1 or 0. But he does not know which is the real one. Then, the next announcement $\varphi ^{n+1}_y$ informs *b* of the $n+1$th bit of *y*, ‘if the *n*th bit of *y* is 1, then the $(n+1)$st bit of *y* is *j*, and if the *n*th bit of *y* is 0, then $(n+1)$st bit of *y* is $1-j$’. We observe that $\theta _n$ successively restricted to the denotation of $\varphi ^{n+1}_x$ and $\varphi ^{n+1}_y$ is a restriction of $\theta _{n+1}$. We can go on in the same way, and successively announce the first *n* bits of both sequences by public announcements in such a way that *a* learns every prefix of *x* and *b* learns every prefix of *y* up to length *n*, as desired; but *a* remains uncertain about every bit in the *y*-prefix that *b* learnt, and *b* remains uncertain about every bit in the *x*-prefix that *a* learnt. For example, given that the agents *a* and *b* only learnt their first bits and that $x = 010000\dots$ and $y =110110\dots$, the next two announcements are now:

$$\begin{aligned} \varphi _x^2= & {} (\lnot \mathbf {x}_1 \rightarrow \mathbf {x}_2) \wedge (\mathbf {x}_1 \rightarrow \lnot \mathbf {x}_2) \\ \varphi _y^2= & {} (\mathbf {y}_1 \rightarrow \mathbf {y}_2) \wedge (\lnot \mathbf {y}_1 \rightarrow \lnot \mathbf {y}_2) \end{aligned}$$

where

$$\begin{aligned} { Int} \left( [\![ \varphi ^2_x]\!]^{\theta _1}\right)= & {} S_{01}\times \{ 0, 1\}^\infty \cup S_{10} \times \{ 0, 1\}^\infty \\ { Int} \left( [\![ \varphi ^2_y]\!]^{\theta _1}\right)= & {} \{ 0, 1\}^\infty \times S_{11} \cup \{ 0, 1\}^\infty \times S_{00}. \end{aligned}$$

Figures 4, 5 and 6 depict the neighbourhood transformations that result from the announcement $\varphi ^2_x$ and, after that, the announcement of $\varphi ^2_y$, consecutively. One can show (details omitted) that

$$\begin{aligned} {\mathcal {M}}, ((x,y), \theta _1)\models & {} \Diamond K_{a} \mathbf {x}_2 \\ {\mathcal {M}}, ((x,y), \theta _1)\models & {} \langle \varphi _x^2\rangle ( K_{a} \mathbf {x}_2 \wedge \lnot (K_{b}\mathbf {x}_2 \vee K_{b} \lnot \mathbf {x}_2)) \\ {\mathcal {M}}, ((x,y), \theta _1)\models & {} \langle \varphi _x^2\rangle \langle \varphi _y^2\rangle (K_{b} \mathbf {y}_2 \wedge \lnot (K_{a}\mathbf {y}_2 \vee K_{a} \lnot \mathbf {y}_2)) \\ {\mathcal {M}}, ((x,y), \theta _2)\models & {} K_{a} \mathbf {x}_2 \end{aligned}$$

We can observe that $\theta _i|\varphi _x^{i+1}|\varphi _y^{i+1}$ is a restriction of $\theta _{i+1}$, as required in this modelling. After every finite sequence of such announcements, *a* knows a prefix of *x* and *b* knows a prefix of *y*, and *a* is uncertain between two dual prefixes of *y* and *b* is uncertain between two prefixes of *x*. So, for example, after 10 announcements, *a* is uncertain whether *y* starts with 110110 or 001001, etc.

## Axiomatization

We now provide the axiomatizations of $${ EL}_{ int} $$, $${ PAL}_{ int} $$, and $${ APAL}_{ int} $$, and prove their soundness and completeness with respect to the proposed semantics.

**Table 1:** The axiomatization $\mathbf {APAL}_{ int}$ [minus (**): $\mathbf {PAL}_{ int}$; and minus additionally, (*): $\mathbf {EL}_{ int}$]

(P)	All instantiations of propositional tautologies	
(*K*-K)	$$K_i(\varphi \rightarrow \psi )\rightarrow (K_i\varphi \rightarrow K_i\psi )$$	
(*K*-T)	$$K_i\varphi \rightarrow \varphi $$	
(*K*-4)	$$K_i\varphi \rightarrow K_iK_i\varphi $$	
(*K*-5)	$$\lnot K_i\varphi \rightarrow K_i\lnot K_i\lnot \varphi $$	
($${ int} $$-K)	$${ int} (\varphi \rightarrow \psi ) \rightarrow ({ int} (\varphi ) \rightarrow { int} (\psi ) )$$	
($${ int} $$-T)	$${ int} (\varphi ) \rightarrow \varphi $$	
($${ int} $$-4)	$${ int} (\varphi ) \rightarrow { int} ({ int} (\varphi ) ) $$	
($$K_{ int} $$)	$$K_i\varphi \rightarrow { int} (\varphi ) $$	
([]-K)	$[\varphi ](\chi \rightarrow \psi )\rightarrow ([\varphi ]\chi \rightarrow [\varphi ]\psi )$	*
(R1)	$[\varphi ]p\leftrightarrow ({ int} (\varphi ) \rightarrow p)$	*
(R2)	$[\varphi ]\lnot \psi \leftrightarrow ({ int} (\varphi ) \rightarrow \lnot [\varphi ]\psi )$	*
(R3)	$[\varphi ](\psi \wedge \chi )\leftrightarrow ([\varphi ]\psi \wedge [\varphi ]\chi )$	*
(R4)	$[\varphi ]{ int} (\psi ) \leftrightarrow ({ int} (\varphi ) \rightarrow { int} ([\varphi ]\psi ) )$	*
(R5)	$[\varphi ]K_i\psi \leftrightarrow ({ int} (\varphi ) \rightarrow K_i[\varphi ]\psi )$	*
(R6)	$[\varphi ][\psi ]\chi \leftrightarrow [\lnot [\varphi ] \lnot { int} (\psi ) ]\chi$	*
(R7)	$\Box \varphi \rightarrow [\chi ]\varphi$ where $\chi \in {\mathcal {L}}_{{ PAL}_{ int} }$	**
(DR1)	From $\varphi$ and $\varphi \rightarrow \psi$, infer $\psi$	
(DR2)	From $\varphi$, infer $K_i\varphi$	
(DR3)	From $\varphi$, infer ${ int} (\varphi )$	
(DR4)	From $\varphi$, infer $[\psi ]\varphi$	*
(DR5)	From $\xi ([\psi ]\chi )$ for all $\psi \in \mathcal {L}_{{ PAL}_{ int} }$, infer $\xi (\Box \chi )$	**

### Definition 22

The axiomatization $\mathbf {APAL}_{ int}$ is given in Table 1. The axiomatization $\mathbf {PAL}_{ int}$ is the one without (DR5) and (R7). We get $\mathbf {EL}_{ int}$ if we further remove axioms (R1)–(R6), ([]-K) and the rule (DR4).^3^

In Table 1, the items (DR1) to (DR5) are the *derivation rules* and the other items are the *axioms*. While the derivation rules (DR1)–(DR4) are standard necessitation rules for the modalities in the language $${\mathcal {L}}_{{ PAL}_{ int} }$$, the rule (DR5) is infinitary. In an infinitary proof system the notion of a derivation is non-standard since a derivation of a formula can involve infinitely many premises; in our system an application of the rule (DR5) requires infinitely many premises. We can think of a *derivation* as a finite-depth tree with possibly infinite branching, where the leaves are axioms or premises, the root is the derived formula, and a step in the tree from child nodes to parent node corresponds to the application of a derivation rule. We write $$\Gamma \vdash \varphi $$ if $$\varphi $$ is derived from a set of formulas $$\Gamma $$ in this way, and $$\vdash \varphi $$ when $$\varphi $$ is derived only from axioms. Note that, due to the infinitary derivation rule (DR5) of $$\mathbf {APAL}_{ int} $$, the set of formulas $$\Gamma $$ deriving $$\varphi $$ within this system can be infinite (see e.g. Rybakov 1997, Chapter 5.4 for a precise treatment of infinitary calculi). We define $${ APAL}_{ int} $$ to be the set of all $$\varphi \in {\mathcal {L}}_{{ APAL}_{ int} }$$ such that $$\vdash \varphi $$. Equivalently, $${ APAL}_{ int} $$ is the smallest subset of $${\mathcal {L}}_{{ APAL}_{ int} }$$ containining the axioms in $$\mathbf {APAL}_{ int} $$ and closed under its derivation rules. An element of $${ APAL}_{ int} $$ is called a *theorem* (of $${ APAL}_{ int} $$). We similarly define the systems $${ EL}_{ int} $$ and $${ PAL}_{ int} $$ from axiomatizations $$\mathbf {EL}_{ int} $$ and $$\mathbf {PAL}_{ int} $$, respectively. However, derivations of $${ EL}_{ int} $$ and $${ PAL}_{ int} $$ are of the form of finite-depth trees with *finite* branching, since $$\mathbf {EL}_{ int} $$ and $$\mathbf {PAL}_{ int} $$ contain only finitary derivation rules.

### Proposition 23

$${ APAL}_{ int} $$ is sound with respect to the class of all topo-models.

### Proof

The soundness of the axiomatization $\mathbf {APAL}_{ int}$ is, as usual, shown by proving that all axioms are validities and that all derivation rules preserve validities. Having proved that, soundness follows by induction on the depth of the derivation tree.

We prove six relevant cases: the first case shows the validity of the reduction axiom for $K_i$, the next two illustrate the need for the constraint in Definition 10.3, the third is concerned with the relation between the knowledge and the interior modalities, and the last two prove validity of the axiom and validity preservation of the inference rule involving the arbitrary announcement modality $\Box$. Let ${\mathcal {M}}=(X, \tau , \Phi , V)$ be a topo-model, $(x, \theta )\in {\mathcal {M}}$ and $\varphi , \psi , \chi \in {\mathcal {L}}_{{ APAL}_{ int} }$.

**(R5)** Suppose $(x, \theta )\models [\varphi ]K_i\psi$. This means that if $(x, \theta )\models { int} (\varphi )$ then $(x, \theta ^\varphi )\models K_i\psi$. Also suppose that $(x, \theta )\models { int} (\varphi )$ and let $z\in \theta (x)(i)$ such that $(z, \theta )\models { int} (\varphi )$, i.e., that $z\in { Int} ([\![ \varphi ]\!]^\theta )$. Then, by assumption, the former implies that $(x, \theta ^\varphi )\models K_i\psi$. In other words, $(y, \theta ^\varphi )\models \psi$ for all $y\in \theta ^\varphi (x)(i)$. Recall, by Definition 13, that $\theta ^\varphi (x)(i)=\theta (x)(i)\cap { Int} ([\![ \varphi ]\!]^\theta )$. Thus, since $z\in \theta (x)(i)\cap { Int} ([\![ \varphi ]\!]^\theta ) =\theta ^\varphi (x)(i)$, we obtain $(z, \theta ^\varphi )\models \psi$ implying that $(z, \theta )\models [\varphi ]\psi$. Since *z* has been chosen arbitrarily from $\theta (x)(i)$, the results holds for every element of $\theta (x)(i)$. Therefore, $(x, \theta )\models K_i[\varphi ]\psi$. Since we also have $(x, \theta )\models { int} (\varphi )$, we conclude $(x, \theta )\models { int} (\varphi ) \rightarrow K_i[\varphi ]\psi$. The converse direction follows similarly.

(**K-4**) Suppose $(x, \theta )\models K_i\varphi$. This means, $(y, \theta )\models \varphi$ for all $y\in \theta (x)(i)$. Let $y\in \theta (x)(i)$ and $z\in \theta (y)(i)$. By Definition 10.3, $\theta (y)(i)=\theta (x)(i)$ and Definition 10.1 guarantees that $\theta (y)(i)\not =\emptyset$. Therefore, by assumption, $(z, \theta )\models \varphi$.

(**K-5**) Suppose $(x, \theta )\models \lnot K_i\varphi$. This means, $(y_0, \theta )\not \models \varphi$ for some $y_0\in \theta (x)(i)$. Let $y\in \theta (x)(i)$. By Definition 10.3, $\theta (x)(i)=\theta (y)(i)$. Therefore, as $y_0\in \theta (y)(i)$ by assumption, we have that there is a $z\in \theta (y)(i)$, namely $z=y_0$, such that $(z, \theta )\not \models \varphi$.

($\mathbf {K}_{ int}$) Suppose $(x, \theta )\models K_i\varphi$. This means, $(y, \theta )\models \varphi$ for all $y\in \theta (x)(i)$. Hence, $\theta (x)(i)\subseteq [\![ \varphi ]\!]^\theta$. By Definition 10, $\theta (x)(i)$ is an open neighbourhood of *x*, therefore we obtain $x\in { Int} [\![ \varphi ]\!]^\theta$, i.e., $(x, \theta )\models { int} (\varphi )$.

**(R7)** Let $\chi \in {\mathcal {L}}_{{ PAL}_{ int} }$ and suppose $(x, \theta )\models \Box \varphi$. By the semantics, we have $(x, \theta )\models \Box \varphi \text{ iff } (\forall \psi \in {\mathcal {L}}_{{ PAL}_{ int} })( (x, \theta )\models [\psi ]\varphi ).$ Therefore, in particular, $(x, \theta )\models [\chi ]\varphi$.

**(DR5)** The proof follows by induction on the complexity of $\xi (\sharp )$.

In case $\xi (\sharp )=\sharp$, we have $\xi ([\psi ]\chi )= [\psi ]\chi$. Suppose $\xi ([\psi ]\chi )$ is valid for all $\psi \in \mathcal {L}_{{ PAL}_{ int} }$. By assumption, we have that $[\psi ]\chi$ is valid for all $\psi \in \mathcal {L}_{{ PAL}_{ int} }$. This implies ${\mathcal {M}}, (x, \theta )\models [\psi ]\chi$ for all $\psi \in \mathcal {L}_{{ PAL}_{ int} }$, all topo-models ${\mathcal {M}}$, and $(x,\theta )\in {\mathcal {M}}$. Therefore, by the semantics, ${\mathcal {M}},(x, \theta )\models \Box \chi$, i.e., ${\mathcal {M}},(x, \theta )\models \xi (\Box \chi )$.

All other, inductive cases are similar, so here we present only the case for $\xi (\sharp )={ int} (\xi ^\prime (\sharp ))$. In this case, we have $\xi ([\psi ]\chi )={ int} (\xi ^\prime ([\psi ]\chi ))$. Suppose that ${ int} (\xi ^\prime ([\psi ]\chi ))$ is valid for all $\psi \in \mathcal {L}_{{ PAL}_{ int} }$. This implies that $\xi ^\prime ([\psi ]\chi )$ is valid for all $\psi \in \mathcal {L}_{{ PAL}_{ int} }$. Otherwise, there is a topo-model ${\mathcal {M}}=(X, \tau , \Phi , V)$ and $(x, \theta )\in {\mathcal {X}}$ such that ${\mathcal {M}}, (x, \theta )\not \models \xi ^\prime ([\psi ]\chi )$ for some $\psi \in \mathcal {L}_{{ PAL}_{ int} }$. This means $x\not \in [\![ \xi ^\prime ([\psi ]\chi )]\!]^\theta$. Since ${ Int} ([\![ \xi ^\prime ([\psi ]\chi )]\!]^\theta ) \subseteq [\![ \xi ^\prime ([\psi ]\chi )]\!]^\theta$, we also obtain that $x\not \in { Int} ([\![ \xi ^\prime ([\psi ]\chi )]\!]^\theta )$, i.e., ${\mathcal {M}}, (x, \theta )\not \models { int} (\xi ^\prime ([\psi ]\chi ))$ contradicting validity of ${ int} (\xi ^\prime ([\psi ]\chi ))$. Then, by IH, we have $\xi ^\prime (\Box \chi )$ valid. This means that $[\![ \xi ^\prime (\Box \chi )]\!]^\theta ={\mathcal {D}}(\theta )$ for all topo-model ${\mathcal {M}}=(X, \tau , \Phi , V)$ and all $\theta \in \Phi$. As ${\mathcal {D}}(\theta )\in \tau$ (by Lemma 12), we have ${\mathcal {D}}(\theta )={ Int} ({\mathcal {D}}(\theta )) ={ Int} ([\![ \xi ^\prime (\Box \chi )]\!]^\theta ) =[\![ { int} (\xi ^\prime (\Box \chi )) ]\!]^\theta$. We can then conclude that ${ int} (\xi ^\prime (\Box \chi ))$ is valid. $\square$

### Corollary 24

$${ EL}_{ int} $$ and $${ PAL}_{ int} $$ are sound with respect to the class of all topo-models.

## Completeness

We now show completeness for $${ EL}_{ int} $$, $${ PAL}_{ int} $$, and $${ APAL}_{ int} $$ with respect to the class of all topo-models. Completeness of $${ EL}_{ int} $$ is shown in a standard way via a canonical model construction and a Truth Lemma that is proved by induction on formula complexity. Completeness for $${ PAL}_{ int} $$ is shown by reducing each formula in $${\mathcal {L}}_{{ PAL}_{ int} }$$ to an equivalent formula of $${\mathcal {L}}_{{ EL}_{ int} }$$. The proof of the completeness for $${ APAL}_{ int} $$ becomes more involved. Reduction axioms for public announcements no longer suffice in the $${ APAL}_{ int} $$ case, and the inductive proof needs a subinduction where announcements are considered. Moreover, the proof system of $${ APAL}_{ int} $$ has an infinitary derivation rule, namely the rule (DR5), and given the requirement of closure under this rule, the maximally consistent sets for that case are defined to be maximally consistent *theories* (see, Sect. 4.2). Lastly, the Truth Lemma requires the more complicated complexity measure on formulas defined in Sect. 2. There, we need to adapt the completeness proof of Balbiani and van Ditmarsch (2015) to our setting.

### Completeness of $${ EL}_{ int} $$ and $${ PAL}_{ int} $$

Let us start with introducing some standard notions used in the completeness proof. These notions can also be found in Blackburn et al. (2001). A set *x* of formulas in ${\mathcal {L}}_{{ EL}_{ int} }$ is called *consistent* if $x\not \vdash \bot$, and *inconsistent* otherwise. A formula $\varphi$ is consistent if $\{\varphi \}$ is consistent. A set of formulas *x* is called *maximally consistent* if *x* is consistent, and any set of formulas properly containing *x* is inconsistent.

We would like to point out that the logic $${ EL}_{ int} $$ is in fact familiar to modal logicians. Its axiomatization consists of the *S*4-type modality $${ int} $$, the *S*5-type modalities $$K_i$$ and the connecting axioms ($$K_{ int} $$). In fact, this axiomatization has been introduced by Goranko and Passy (1992) in a more general way as an extension of normal modal logics with the global modality, where our ($$K_{ int} $$) plays the role of the so-call “inclusion” axiom scheme. As also studied in Blackburn et al. (2001, Chapter 7.1), from the syntactic point of view, the system $${ EL}_{ int} $$ can be treated as a normal multi-modal logic. Therefore, proofs of Lemma 25 and Lemma 26 (below) are standard (see, e.g. Proposition 4.16 and Lemma 4.17 in Blackburn et al. 2001, p. 199, respectively).

#### Lemma 25

For any maximally consistent set *x* of formulas in $${ EL}_{ int} $$:

1. *x* is closed under (DR1),
2. $${ EL}_{ int} \subseteq x$$,
3. for all formulas $\varphi \in {\mathcal {L}}_{{ EL}_{ int} }$, $\varphi \in x$ or $\lnot \varphi \in x$,
4. for all formulas $\varphi , \psi \in {\mathcal {L}}_{{ EL}_{ int} }$, $\varphi \wedge \psi \in x$ iff $\varphi \in x$ and $\psi \in x$.

Let $$X^c$$ be the set of all maximally consistent sets of $${ EL}_{ int} $$. We define relations $$\sim _i$$ on $$X^c$$ as $$x\sim _i y \ \text{ iff } \ \forall \varphi \in \mathcal {L}_{{ EL}_{ int} }(K_i\varphi \in x \ \text{ iff } \ K_i\varphi \in y)$$. Notice that the latter is equivalent to: $$\forall \varphi \in \mathcal {L}_{{ EL}_{ int} }(K_i\varphi \in x \ \text{ implies } \ \varphi \in y)$$ since $$K_i$$ is an *S*5 modality. As each $$K_i$$ is of *S*5 type, every $$\sim _i$$ is an equivalence relation, hence, it induces equivalence classes on $$X^c$$. Let $$[x]_i$$ denote the equivalence class of *x* induced by the relation $$\sim _i$$. Moreover, we define $$\widehat{\varphi }=\{y\in X^c \ | \ \varphi \in y\}$$. Observe that $$x\in \widehat{\varphi }$$ iff $$\varphi \in x$$.

#### Lemma 26

(Lindenbaum’s Lemma) Each consistent set can be extended to a maximally consistent set.

#### Definition 27

We define the canonical model ${\mathcal {X}}^c=(X^c, \tau ^c, \Phi ^c, V^c)$ as follows:

- $$X^c$$ is the set of all maximally consistent sets of $${ EL}_{ int} $$;
- $\tau ^c$ is the topological space generated by the subbase
  $$\begin{aligned} \Sigma =\{[x]_i\cap \widehat{{ int} (\varphi ) } \ | \ x\in X^c, \varphi \in \mathcal {L}_{{ EL}_{ int} }\quad \text{ and }\quad i\in {\mathcal {A}}\}; \end{aligned}$$
- $$x\in V^c(p) \ \text{ iff } \ p\in x, \ \text{ for } \text{ all } \ p\in Prop $$;
- $\Phi ^c=\{\theta ^c|_U\;|\;U\in \tau ^c\}$, where we define $\theta ^c:X^c\rightarrow {\mathcal {A}}\rightarrow \tau ^c$ as $\theta ^c(x)(i)=[x]_i$, for $x\in X^c$ and $i\in {\mathcal {A}}$.

We first need to show that $(X^c, \tau ^c, \Phi ^c)$ is indeed a topo-frame.

#### Lemma 28

$(X^c, \tau ^c, \Phi ^c)$ is a topo-frame.

#### Proof

In order to show the above statement, we need to show that $(X^c, \tau ^c)$ is a topological space, and $\Phi ^c$ satisfies the conditions in Definition 10. For the former, we only need to show that $\Sigma$ covers $X^c$, i.e., that $\bigcup \Sigma =X^c$, since $\tau ^c$ is generated by a *subbase*, namely by $\Sigma$ (in the way described in Sect. 2.2). Since every element of $\Sigma$ is a subset of $X^c$, we obviously have $\bigcup \Sigma \subseteq X^c$. Observe moreover that, since $\widehat{{ int} (\top ) } =X^c$, we have $[x]_i\cap \widehat{{ int} (\top ) } =[x]_i\in \Sigma$ for each $x\in X^c$ and $i\in {\mathcal {A}}$. Now let $x\in X^c$. Since every $\sim _i$ is an equivalence relation, in particular, each $\sim _i$ is reflexive, we have $x\in [x]_i$. Therefore, we obtain $\bigcup _{x\in X^c}[x]_i =X^c\subseteq \bigcup \Sigma$ for any $i\in {\mathcal {A}}$. Hence, we conclude $\bigcup \Sigma =X^c$ implying that $(X^c, \tau ^c)$ is a topological space. We now show that $\Phi ^c$ satisfies the conditions in Definition 10. Let $\theta \in \Phi ^c$. Thus, by definition of $\Phi ^c$, we have $\theta =\theta ^c|_U$ for some $U\in \tau ^c$ (in particular, note that $\theta ^c=\theta ^c|_{X^c}$). Therefore, we have that ${\mathcal {D}}(\theta )={\mathcal {D}}(\theta ^c)\cap U=X^c\cap U=U\subseteq X^c$ and $\theta (x)(i)=\theta ^c(x)(i)\cap U=[x]_i\cap U$ for any $x\in {\mathcal {D}}(\theta )$ and $i\in {\mathcal {A}}$. As argued above, $[x]_i\in \Sigma$ for all $x\in X^c$ and each $i\in {\mathcal {A}}$. We therefore obtain that function $\theta$ is defined as a partial function such that $\theta : X^c\rightharpoonup {\mathcal {A}}\rightarrow \tau ^c$. For condition (1), let $x\in {\mathcal {D}}(\theta )$. Since ${\mathcal {D}}(\theta )=U$ and $\theta (x)(i)=[x]_i\cap U$, we also have $x\in [x]_i\cap U=\theta (x)(i)$ for all $i\in {\mathcal {A}}$. Moreover, since $\theta (x)(i)=[x]_i\cap U\subseteq U={\mathcal {D}}(\theta )$, we also satisfy condition (2). For condition (3), let $y\in \theta (x)(i)$. As $\theta (x)(i)=[x]_i\cap U$, we have $y\in [x]_i$ and $y\in {\mathcal {D}}(\theta )$. While the latter proves the first consequent of condition (3), the former implies $[y]_i=[x]_i$ since $[x]_i$ is an equivalence class. We therefore obtain $\theta (y)(i)=[y]_i\cap U=[x]_i\cap U=\theta (x)(i)$. Condition (4) is satisfied by definition of $\Phi ^c$. $\square$

#### Lemma 29

(Truth Lemma) For every $\varphi \in \mathcal {L}_{{ EL}_{ int} }$ and for each $x\in X^c$, $\varphi \in x \ \text{ iff } \ {\mathcal {X}}^c, (x, \theta ^c)\models \varphi .$

#### Proof

The case for the propositional variables follows from the definition of $$V^c$$ and the cases for the Booleans are straightforward. We only show the cases for $$K_i$$ and $${ int} $$.

**Case** $$\varphi = K_i\psi $$

($\Rightarrow$) Suppose $K_i\psi \in x$ and let $y\in \theta ^c(x)(i)$. Since $y\in \theta ^c(x)(i)=[x]_i$, by definition of $\sim _i$, we have $K_i\psi \in y$. Then, by T-axiom for $K_i$, we obtain $\psi \in y$. Then, by IH, ${\mathcal {X}}^c, (y, \theta ^c)\models \psi$. Therefore ${\mathcal {X}}^c, (x, \theta ^c)\models K_i\psi$.

($\Leftarrow$) Suppose $K_i\psi \not \in x$. Then, $\{K_i\gamma \ | \ K_i\gamma \in x\}\cup \{\lnot \psi \}$ is a consistent set. We can then extend it to a maximally consistent set *y*. As $\{K_i\gamma \ | \ K_i\gamma \in x\}\subseteq y$, we have $y\in [x]_i$ meaning that $y\in \theta ^c(x)(i)$. Moreover, since $\lnot \psi \in y$, $\psi \not \in y$. Therefore, we have a maximally consistent set $y\in \theta ^c(x)(i)$ such that $\psi \not \in y$. By (IH), ${\mathcal {X}}^c, (y, \theta ^c)\not \models \psi$. Hence, ${\mathcal {X}}^c, (x, \theta ^c)\not \models K_i\psi$.

**Case** $$\varphi = { int} (\psi ) $$

($$\Rightarrow $$) Suppose $${ int} (\psi ) \in x$$. Consider the set $$[x]_i\cap \widehat{{ int} (\psi ) } $$ for some $$i\in {\mathcal {A}}$$. Obviously, $$x\in [x]_i\cap \widehat{{ int} (\psi ) } $$ and $$[x]_i\cap \widehat{{ int} (\psi ) } $$ is open (since it is in $$\Sigma $$). Now let $$y\in [x]_i\cap \widehat{{ int} (\psi ) } $$. Since $$y\in \widehat{{ int} (\psi ) } $$, $${ int} (\psi ) \in y$$. Then, by ($${ int} $$ -T), since *y* is maximal consistent, we have $$\psi \in y$$. Thus, by IH, we have $$(y, \theta ^c)\models \psi $$. Therefore, $$y\in [\![ \psi ]\!]^{\theta ^c}$$. This implies $$[x]_i\cap \widehat{{ int} (\psi ) } \subseteq [\![ \psi ]\!]^{\theta ^c}$$. And, since $$x\in [x]_i\cap \widehat{{ int} (\psi ) } \in \tau ^c$$, we have $$ x\in { Int} [\![ \psi ]\!]^{\theta ^c} $$, i.e., $$(x, \theta ^c)\models { int} (\psi ) $$.

($\Leftarrow$) Suppose $(x, \theta ^c)\models { int} (\psi )$, i.e., $x\in { Int} [\![ \psi ]\!]^{\theta ^c}$. Recall that the set of finite intersections of the elements of $\Sigma$ forms a base, which we denote by ${\mathcal {B}}_\Sigma$, for $\tau ^c$. $x\in { Int} [\![ \psi ]\!]^{\theta ^c}$ implies that there exists an open $U\in {\mathcal {B}}_\Sigma$ such that $x\in U\subseteq [\![ \psi ]\!]^{\theta ^c}$. Given the construction of ${\mathcal {B}}_\Sigma$, *U* is of the form

$$\begin{aligned} U=\bigcap _{i\in I_1} [x_1]_i \cap \cdots \bigcap _{i\in I_n}[x_k]_i \cap \bigcap _{\eta \in \mathrm {Form_{fin}}} \widehat{{ int} (\eta ) } \end{aligned}$$

where $$I_1, \ldots , I_n$$ are finite subsets of $${\mathcal {A}}$$, $$x_1,\ldots ,x_k\in X^c$$ and $${\mathrm {Form}}_{\mathrm{fin}}$$ is a finite subset of $${\mathcal {L}}_{{ EL}_{ int} }$$. Since $${ int} $$ is a normal modality, we can simply write

$$\begin{aligned} U= \bigcap _{i\in I_1} [x_1]_i \cap \cdots \bigcap _{i\in I_n}[x_k]_i \cap \widehat{{ int} (\gamma ) } , \end{aligned}$$

where $\bigwedge _{\eta \in \mathrm {Form_{fin}}} \eta :{=} \gamma$. Since *x* is in each $[x_j]_i$ with $1\le j\le k$, we have $[x_j]_i=[x]_i$ for all such *j*. Therefore, we have

$$\begin{aligned} x\in U= (\bigcap _{i\in I}[x]_i) \cap \widehat{{ int} (\gamma ) } \subseteq [\![ \psi ]\!]^{\theta ^c}, \end{aligned}$$

where $$I= I_1\cup \cdots \cup I_n$$.

This implies, for all $y\in (\bigcap _{i\in I}[x]_i)$, if $y\in \widehat{{ int} (\gamma ) }$ then $\psi \in y$. From this, we can say $\bigcup _{i\in I}\{K_i\sigma \ | \ K_i\sigma \in x\}\vdash { int} (\gamma ) \rightarrow \psi$. Then, there is a finite subset $\Gamma \subseteq \bigcup _{i\in I}\{K_i\sigma \ | \ K_i\sigma \in x\}$ such that $\vdash \bigwedge _{\lambda \in \Gamma } \lambda \rightarrow ({ int} (\gamma ) \rightarrow \psi )$. It then follows:

$$\begin{aligned} \begin{array}{ll} 1. \vdash { int} (\bigwedge _{\lambda \in \Gamma } \lambda \rightarrow ({ int} (\gamma ) \rightarrow \psi )) &{}\quad (\text{ DR3 })\\ 2. \vdash { int} (\bigwedge _{\lambda \in \Gamma } \lambda ) \rightarrow { int} ({ int} (\gamma ) \rightarrow \psi ) ) &{}\quad ({ int} \text{-K })~\text{ and }~(\text{ DR1 })\\ 3. \vdash (\bigwedge _{\lambda \in \Gamma }{ int} (\lambda ) ) \rightarrow { int} ({ int} (\gamma ) \rightarrow \psi ) ) &{}\quad ({ int} \text{-K }) \end{array} \end{aligned}$$

Observe that each $$\lambda \in \Gamma $$ is of the form $$K_j\alpha $$ for some $$K_j\alpha \in \bigcup _{i\in I}\{K_i\sigma \ | \ K_i\sigma \in x\}$$ and we have $$\vdash K_i\varphi \leftrightarrow { int} (K_i\varphi ) $$. Therefore, $$\vdash (\bigwedge _{\lambda \in \Gamma }\lambda ) \rightarrow { int} ({ int} (\gamma ) \rightarrow \psi ) )$$. Thus, since $$\bigwedge _{\lambda \in \Gamma }\lambda \in x$$ (by $$\Gamma \subseteq x$$), we have $${ int} ({ int} (\gamma ) \rightarrow \psi ) )\in x$$. Then, by ($${ int} $$-K), (DR1) and since $$\vdash { int} ({ int} (\gamma ) ) \leftrightarrow { int} (\gamma ) $$ and $$x\in \widehat{{ int} (\gamma ) }$$ (i.e., $${ int} (\gamma ) \in x$$), we obtain $${ int} (\psi ) \in x$$. $$\square $$

Our canonical model construction is similar to the one for the single-agent case in Bjorndahl (2017). We give a comparison in Sect. 6.

#### Theorem 30

$${ EL}_{ int} $$ is complete with respect to the class of all topo-models.

#### Theorem 31

$${ PAL}_{ int} $$ is complete with respect to the class of all topo-models.

#### Proof

This follows from Theorem 30 by reduction in a standard way: using the size measure $$S(\varphi )$$ of Definition 3 for the language $$\mathcal {L}_{{ PAL}_{ int} }$$ provides the desired result via Lemma 7 (note that the strict orders $$<^S$$ and $$<^S_d $$ given in Definition 5 are equivalent on the language $$\mathcal {L}_{{ PAL}_{ int} }$$). We refer to van Ditmarsch et al. (2007, Chapter 7.4) for a detailed presentation of the completeness method via reduction, and in particular to Wang and Cao (2013, Theorem 10, p. 111) for an analogous proof. A similar proof for single-agent $${ EL}_{ int} $$ is also presented in Bjorndahl (2017, Section 4).

### Completeness of $${ APAL}_{ int} $$

We now reuse the technique of Balbiani and van Ditmarsch (2015) in the setting of topological semantics. Given the closure requirement under derivation rule (DR5) it seems more proper to call maximally consistent sets of $${ APAL}_{ int} $$ maximally consistent theories, as further explained below.

#### Definition 32

A set *x* of formulas is called a *theory* iff ${ APAL}_{ int} \subseteq x$ and *x* is closed under (DR1) and (DR5). A theory *x* is said to be consistent iff $\bot \not \in x$. A theory *x* is maximally consistent iff *x* is consistent and any set of formulas properly containing *x* is inconsistent.

The set $${ APAL}_{ int} $$ constitutes the smallest theory. Moreover, maximally consistent theories of $${ APAL}_{ int} $$ posses the usual properties of maximally consistent sets:

#### Proposition 33

For any maximally consistent theory *x*, $\varphi \not \in x$ iff $\lnot \varphi \in x$, and $\varphi \wedge \psi \in x$ iff $\varphi \in x$ and $\psi \in x$.

In the setting of our axiomatization based on the infinitary rule (DR5), we will say that a set *x* of formulas is consistent iff there exists a consistent theory *y* such that $x\subseteq y$. Obviously, maximal consistent theories are maximal consistent sets of formulas. Under the given definition of consistency for sets of formulas, maximal consistent sets of formulas are also maximal consistent theories.

#### Definition 34

Let $\varphi \in {\mathcal {L}}_{{ APAL}_{ int} }$ and $i \in {\mathcal {A}}$. Then $x+\varphi :{=} \{\psi \ | \ \varphi \rightarrow \psi \in x\}$, $K_i x :{=}\{\varphi \ | \ K_i\varphi \in x\}$, and ${ int} (x) :{=}\{\varphi \ | \ { int} (\varphi ) \in x\}$.

#### Lemma 35

For any theory *x* of $${ APAL}_{ int} $$ and $$\varphi \in {\mathcal {L}}_{{ APAL}_{ int} }$$,

1. $x+\varphi$ is a theory that contains *x* and $\varphi$,
2. $$K_ix$$ is a theory,
3. $${ int} (x) $$ is a theory, and
4. $${ int} (x) \subseteq x$$.

#### Proof

Follows in a similar way as in the proof of Balbiani et al. (2008, Lemma 4.11), and here we only prove items 3 and 4. Suppose *x* is a theory of $${ APAL}_{ int} $$ and $$\varphi \in \mathcal {L}_{{ APAL}_{ int} }$$.

3. Suppose $$\varphi \in { APAL}_{ int} $$. Since $$\varphi $$ is a theorem, by (DR3), $${ int} (\varphi ) $$ is a theorem of $${ APAL}_{ int} $$ as well. Therefore, $${ int} (\varphi ) \in x$$ meaning that $$\varphi \in { int} (x) $$. Hence, $${ APAL}_{ int} \subseteq { int} (x) $$. Let us now show that $${ int} (x) $$ is closed under (DR1). Suppose $$\varphi , \varphi \rightarrow \psi \in { int} (x) $$. This means, by definition of $${ int} (x) $$, that $${ int} (\varphi ) , { int} (\varphi \rightarrow \psi ) \in x$$. By ($${ int} $$-K) and *x* being closed under (DR1), we obtain $${ int} (\psi ) \in x$$, i.e., $$\psi \in { int} (x) $$. Finally we show that $${ int} (x) $$ is closed under (DR5). Let $$\xi ([\psi ]\chi )\in { int} (x) $$ for all $$\psi \in { PAL}_{ int} $$. This means $${ int} (\xi ([\psi ]\chi )) \in x$$ for all $$\psi \in { PAL}_{ int} $$. As $${ int} (\xi ([\psi ]\chi )) $$ is also a necessity form and *x* is closed under (DR5), $${ int} (\xi (\Box \chi )) \in x$$ meaning that $$\xi (\Box \chi )\in { int} (x) $$. We therefore conclude that $${ int} (x) $$ is a theory.
4. Suppose $$\varphi \in { int} (x) $$. This means $${ int} (\varphi ) \in x$$. Therefore, by ($${ int} $$-T) and (DR1), we obtain $$\varphi \in x$$. As $$\varphi $$ has been taken arbitrarily from $${ int} (x) $$, we conclude that $${ int} (x) \subseteq x$$.

#### Lemma 36

Let $\varphi \in \mathcal {L}_{{ APAL}_{ int} }$. For all theories *x*, $x+\varphi$ is consistent iff $\lnot \varphi \not \in x$.

#### Proof

Let $\varphi \in \mathcal {L}_{{ APAL}_{ int} }$ and *x* be a theory. Then $\lnot \varphi \in x$ iff $\varphi \rightarrow \bot \in x$ (as $\lnot \varphi \leftrightarrow \varphi \rightarrow \bot$ is a theorem) iff $\bot \in x + \varphi$. Therefore, $x+\varphi$ is inconsistent iff $\lnot \varphi \in x$, i.e., $x+\varphi$ is consistent iff $\lnot \varphi \not \in x$. $\square$

#### Lemma 37

(Lindenbaum’s Lemma Balbiani et al. 2008) Each consistent theory can be extended to a maximal consistent theory.

#### Lemma 38

If $K_i\varphi \not \in x$, then there is a maximally consistent theory *y* such that $K_ix\subseteq y$ and $\varphi \not \in y$.

#### Proof

Let $\varphi \in \mathcal {L}_{{ APAL}_{ int} }$ and *x* be such that $K_i\varphi \not \in x$. Thus, $\varphi \not \in K_ix$. Hence, by Lemma 36, $K_ix+\lnot \varphi$ is consistent. Then, by Lemma 37, there exists a maximally consistent set *y* such that $K_ix+\lnot \varphi \subseteq y$. Therefore $K_i x\subseteq y$ and $\varphi \not \in y$. $\square$

#### Lemma 39

For all $\varphi \in {\mathcal {L}}_{{ APAL}_{ int} }$ and all maximally consistent theories *x*, $\Box \varphi \in x$ iff for all $\psi \in {\mathcal {L}}_{{ PAL}_{ int} }, [\psi ]\varphi \in x$.

#### Proof

Let $\varphi \in {\mathcal {L}}_{{ APAL}_{ int} }$ and *x* be a maximally consistent theory.

($\Rightarrow$) Suppose $\Box \varphi \in x$. Then, by (R7) and (DR1), we have $[\psi ]\varphi \in x$ for all $\psi \in {\mathcal {L}}_{{ PAL}_{ int} }$.

($\Leftarrow$) Suppose $[\psi ]\varphi \in x$ for all $\psi \in {\mathcal {L}}_{{ PAL}_{ int} }$. Consider the necessity form $\sharp$. By assumption, $\sharp ([\psi ]\varphi )$ for all $\psi \in {\mathcal {L}}_{{ PAL}_{ int} }$. Then, since *x* is closed under (DR5), $\sharp (\Box \varphi )\in x$, i.e., $\Box \varphi \in x$ as well. $\square$

The definition of *the canonical model for*$${ APAL}_{ int} $$ is the same as for $${ EL}_{ int} $$, except that the maximally consistent sets are maximally consistent theories of $${ APAL}_{ int} $$. We now come to the Truth Lemma for the logic $${ APAL}_{ int} $$. Here we use the complexity measure $$\psi <^S_d \varphi $$, and we recall that $$\theta ^c:X^c\rightarrow {\mathcal {A}}\rightarrow \tau ^c$$ is defined as $$\theta ^c(x)(i)=[x]_i$$, for $$x\in X^c$$ and $$i\in {\mathcal {A}}$$.

#### Lemma 40

(Truth Lemma) For every $\varphi \in \mathcal {L}_{{ APAL}_{ int} }$ and for each $x\in X^c$, $\varphi \in x \ \text{ iff } \ {\mathcal {X}}^c, (x, \theta ^c)\models \varphi$.

#### Proof

Let $$\varphi \in \mathcal {L}_{{ APAL}_{ int} }$$ and $$x\in {\mathcal {X}}^c$$. The proof is by $$<^S_d $$-induction on $$\varphi $$, where the case $$\varphi = [\psi ]\chi $$ is proved by a subinduction on $$\chi $$. We therefore consider 13 cases.

**Case**$$\varphi = p$$

$$\begin{aligned} \begin{array}{lll} x\in p &{}\quad \text{ iff } &{}\quad x\in V^c(p)\\ &{}\quad \text{ iff } &{}\quad (x, \theta ^c)\models p \end{array} \end{aligned}$$

**Induction Hypothesis (IH)**: For all formulas $\psi \in {\mathcal {L}}_{{ APAL}_{ int} }$, if $\psi <^S_d \varphi$, then $\psi \in x \ \text{ iff } \ {\mathcal {X}}^c, (x, \theta ^c)\models \psi$.

The cases negation and conjunction are as in Truth Lemma 29 for ${ EL}_{int}$, where we observe that the subformula order is subsumed under the $<^S_d$ order (see Lemma 6.2). We proceed with the knowledge and interior modalities, i.e., cases $\varphi =K_i\psi$ and $\varphi ={ int} (\psi )$ respectively, and then with the subinduction on $\chi$ for case announcement $\varphi = [\psi ]\chi$, and finally with the case $\varphi =\Box \psi$.

**Case** $$\varphi = K_i\psi $$

For the direction from left-to-right, see Truth Lemma 29. For ($\Leftarrow$), suppose $K_i\psi \not \in x$. Then, by Lemma 38, there exists a maximally consistent theory *y* such that $K_ix\subseteq y$ and $\psi \not \in y$. By $\psi <^S_d K_i\psi$ and (IH), $(y, \theta ^c)\not \models \psi$. Since $K_ix\subseteq y$, we have $y\in [x]_i$ meaning that $y\in \theta ^c(x)(i)$. Therefore, by the semantics, ${\mathcal {X}}^c, (x, \theta ^c)\not \models K_i\psi$.

**Case** $$\varphi = { int} (\psi ) $$

For the direction from left-to-right, see Truth Lemma 29. For ($\Leftarrow$), suppose ${ int} (\psi ) \not \in x$. We want to show that $x\not \in { Int} ([\![ \psi ]\!]^{\theta ^c})$, i.e., show that for all $U\in {\mathcal {B}}_\Sigma$ with $x\in U$, we obtain $U\not \subseteq [\![ \psi ]\!]^{\theta ^c}$, where ${\mathcal {B}}_\Sigma$ is the base of ${\mathcal {X}}^c$ constructed by closing $\Sigma$ under finite intersections (as in the proof of Truth Lemma 29). Let $U\in {\mathcal {B}}_\Sigma$ such that $x\in U$. Given the construction of ${\mathcal {B}}_\Sigma$, *U* is of the form

$$\begin{aligned} U=\left( \bigcap _{i\in I}[x]_i\right) \cap \widehat{{ int} (\gamma ) } , \end{aligned}$$

where *I* and ${ int} (\gamma )$ are as in Truth Lemma 29. In order to complete the proof, we construct a maximally consistent theory $y\in U$ such that $y\not \in [\![ \psi ]\!]^{\theta ^c}$. Therefore, this maximally consistent theory *y* should satisfy the following properties:

1. $$\bigcup _{i\in I} \{K_i\sigma \ | \ K_i\sigma \in x\}\subseteq y$$, i.e., $$y\in \bigcap _{i\in I}[x]_i$$,
2. $${ int} (\gamma ) \in y$$, i.e., $$y\in \widehat{{ int} (\gamma ) } $$,
3. $$\lnot \psi \in y$$, or equivalently, $$\psi \not \in y$$.

Toward the goal of finding this maximal consistent *y*, we first construct a consistent theory *z* (that we then later expand to the maximal consistent theory *y*). Consider the set of formulas

$$\begin{aligned} z_0:{=}\bigcup _{i\in I} \{K_i\sigma \ | \ K_i\sigma \in x\}\cup \{ { int} (\gamma ) \}\cup { APAL}_{ int} , \end{aligned}$$

and close $$z_0$$ under (DR1) and (DR5) to obtain *z*. It is guaranteed that *z* is a theory since it includes $$ { APAL}_{ int} $$ and it is closed under (DR1) and (DR5). Moreover, $$z_0\subseteq x$$, since (1) $$\bigcup _{i\in I} \{K_i\sigma \ | \ K_i\sigma \in x\}\subseteq x$$ and (2) $${ int} (\gamma ) \in x$$ because $$x\in U=(\bigcap _{i\in I}[x]_i) \cap \widehat{{ int} (\gamma ) } $$, and thus, $$x\in \widehat{{ int} (\gamma ) } $$. Therefore, $$z_0\subseteq x$$ and since *z* is the smallest theory containing $$z_0$$ (by construction), we obtain $$z\subseteq x$$. It follows that *z* is consistent since *x* is consistent, being a maximally consistent theory. We now consider the set $${ int} (z) $$. Similarly, $${ int} (z) $$ is a consistent theory such that $${ int} (z) \subseteq z\subseteq x$$ (by Lemma 35.3–4 and *x* being a maximally consistent theory). Furthermore, $$\bigcup _{i\in I} \{K_i\sigma \ | \ K_i\sigma \in x\}\cup \{ { int} (\gamma ) \}\subseteq { int} (z) $$, since $$\vdash K_i\sigma \leftrightarrow { int} (K_i\sigma ) $$ and $$K_i\sigma \in z$$ for each $$i\in I$$, and similarly since $$\vdash { int} (\gamma ) \leftrightarrow { int} ({ int} (\gamma ) ) $$ and $${ int} (\gamma ) \in z$$. In fact, given that *z* is the smallest theory constructed from $$z_0$$ by closing $$z_0$$ under (DR1) and (DR5) and $${ int} (z) $$ is also a consistent theory such that $$z_0\subseteq { int} (z) \subseteq z$$, we obtain $${ int} (z) =z$$. Observe that, since $${ int} (\psi ) \not \in x$$ and $$z\subseteq x$$, we have $${ int} (\psi ) \not \in z$$. Therefore, the fact that $${ int} (\psi ) \not \in { int} (z) =z$$ implies that $$\psi \not \in z$$. Finally, we extend the consistent theory *z* to the set of formulas $$z+\lnot \psi $$. By Lemma 35.1, we know that $$z+\lnot \psi $$ is a theory such that $$z\subseteq z+\lnot \psi $$ and $$\lnot \psi \in z+\lnot \psi $$. Moreover, since $$\psi \not \in z$$, Lemma 36 implies that $$z+\lnot \psi $$ is a consistent theory. Thus, by Lemma 37, there exists a maximally consistent set *y* such that $$z+\lnot \psi \subseteq y$$. Hence, we have a maximally consistent set *y* such that:

1. $$\bigcup _{i\in I} \{K_i\sigma \ | \ K_i\sigma \in x\}\subseteq y$$, since $$\bigcup _{i\in I} \{K_i\sigma \ | \ K_i\sigma \in x\}\subseteq z\subseteq y$$,
2. $${ int} (\gamma ) \in y$$, since $${ int} (\gamma ) \in z\subseteq y$$, and
3. $$\lnot \psi \in y$$, since $$\lnot \psi \in z+\lnot \psi \subseteq y$$.

Therefore, $y\in (\bigcap _{i\in I}[x]_i) \cap \widehat{{ int} (\gamma ) } =U$ (by (1) and (2)) such that $y\not \in [\![ \psi ]\!]^{\theta ^c}$ (by IH)). Thus, $U\not \subseteq [\![ \psi ]\!]^{\theta ^c}$ implying that $x\not \in { Int} ([\![ \psi ]\!]^{\theta ^c})$.

**Case**$$\varphi = [\psi ] p$$

$$\begin{aligned} \begin{array}{llllll} [\psi ]p\in x &{}\quad \text{ iff }&{}\quad { int} (\psi ) \rightarrow p \in x &{}\quad \text {(R1)} \\ &{}\quad \text{ iff }&{}\quad (x, \theta ^c)\models { int} (\psi ) \rightarrow p &{}\quad \text{((IH) } \text{ and } \text{ Lemma } \text{7.1) } \\ &{}\quad \text{ iff }&{}\quad (x, \theta ^c)\models [\psi ]p &{}\quad \text{(R1) } \\ \end{array} \end{aligned}$$

**Case**$\varphi :{=} [\psi ] \lnot \eta$ Use (R2) and (IH) and, by Lemma 7.2, ${ int} (\psi ) \rightarrow \lnot [\psi ]\eta <^S_d [\psi ]\lnot \eta$.

**Case**$\varphi :{=} [\psi ] (\eta \wedge \sigma )$ Use (R3) and (IH) and, by Lemma 7.3, $[\psi ]\eta \wedge [\psi ]\sigma <^S_d [\psi ] (\eta \wedge \sigma )$.

**Case**$\varphi :{=} [\psi ] { int} (\eta )$ Use (R4) and (IH) and, by Lemma 7.4, ${ int} (\psi ) \rightarrow { int} ( [\psi ]\eta ) <^S_d [\psi ] { int} (\eta )$.

**Case**$\varphi :{=} [\psi ] K_i\eta$ Use (R5) and (IH) and, by Lemma 7.5, ${ int} (\psi ) \rightarrow K_i [\psi ]\eta <^S_d [\psi ] K_i\eta$.

**Case**$\varphi :{=} [\psi ] [\eta ]\sigma$ Use (R6) and (IH) and, by Lemma 7.6, $[\lnot [\psi ]\lnot { int} (\eta ) ]\sigma <^S_d [\psi ][\eta ]\sigma$.

**Case**$\varphi :{=} [\psi ] \Box \sigma$ For all $\eta \in {\mathcal {L}}_{{ PAL}_{ int} }$, $[\psi ][\eta ]\sigma <^S_d [\psi ] \Box \sigma$, as $[\psi ] \Box \sigma$ has one more $\Box$ than $[\psi ][\eta ]\sigma$. Therefore, it suffices to show $[\psi ] \Box \sigma \in x \ \text{ iff } \ \forall \eta \in {\mathcal {L}}_{{ PAL}_{ int} }, [\psi ][\eta ]\sigma \in x.$

($\Leftarrow$) Consider the necessity form $[\psi ]\sharp$ and assume that for all $\eta \in {\mathcal {L}}_{{ PAL}_{ int} }$, $[\psi ][\eta ]\sigma \in x$, i.e., for all $\eta \in {\mathcal {L}}_{{ PAL}_{ int} }$, $[\psi ]\sharp ([\eta ]\sigma )\in x$. As *x* is closed under (DR5), we obtain $[\psi ]\sharp (\Box \sigma )\in x$, i.e., $[\psi ]\Box \sigma \in x$.

($\Rightarrow$) Suppose $[\psi ]\Box \sigma \in x$. We have

$$\begin{aligned} \begin{array}{ll} \vdash \Box \sigma \rightarrow [\eta ]\sigma , \ \text{ for } \text{ all } \ \eta \in {\mathcal {L}}_{{ PAL}_{ int} } &{}\quad \text{(R7) }\\ \vdash [\psi ](\Box \sigma \rightarrow [\eta ]\sigma ) \ \text{ for } \text{ all } \ \eta \in {\mathcal {L}}_{{ PAL}_{ int} } &{}\quad \text{(DR4) }\\ \vdash [\psi ]\Box \sigma \rightarrow [\psi ][\eta ]\sigma , \ \text{ for } \text{ all } \ \eta \in {\mathcal {L}}_{{ PAL}_{ int} } &{}\quad ([]\text{-K }),~\text{(DR1) } \end{array} \end{aligned}$$

Therefore, for all $\eta \in {\mathcal {L}}_{{ PAL}_{ int} }, [\psi ][\eta ]\sigma \in x$. As $[\psi ][\eta ]\sigma <^S_d [\psi ] \Box \sigma$ for all $\eta \in {\mathcal {L}}_{{ PAL}_{ int} }$, by (IH), we have for all $\eta \in {\mathcal {L}}_{{ PAL}_{ int} }, (x, \theta ^c)\models [\psi ][\eta ]\sigma$. We then obtain

$$\begin{aligned} \begin{array}{ll} &{}\quad (\forall \eta \in {\mathcal {L}}_{{ PAL}_{ int} }) (x, \theta ^c)\models [\psi ][\eta ]\sigma \\ \text{ iff } &{}\quad (\forall \eta \in {\mathcal {L}}_{{ PAL}_{ int} })((x, \theta ^c)\models { int} (\psi ) \ \text{ implies } \ (x, (\theta ^c)^\psi )\models [\eta ]\sigma ) \\ \text{ iff } &{}\quad (x, \theta ^c)\models { int} (\psi ) \ \text{ implies } \ (\forall \eta \in {\mathcal {L}}_{{ PAL}_{ int} })( (x, (\theta ^c)^\psi )\models [\eta ]\sigma ) \\ \text{ iff } &{}\quad (x, \theta ^c)\models { int} (\psi ) \ \text{ implies } \ (x, (\theta ^c)^\psi )\models \Box \sigma \\ \text{ iff } &{}\quad (x, \theta ^c)\models [\psi ] \Box \sigma \\ \end{array} \end{aligned}$$

**Case**$\varphi :{=} \Box \psi$ Again note that for all $\eta \in {\mathcal {L}}_{{ PAL}_{ int} }$, $[\eta ]\psi <^S_d \Box \psi$, as $\Box \psi$ has one more $\Box$ than $[\eta ]\psi$ (see Lemmas 6.4 and 6.5). Therefore, we obtain

$$\begin{aligned} \begin{array}{llll} \Box \psi \in x &{}\quad \text{ iff }&{}\quad (\forall \eta \in {\mathcal {L}}_{{ PAL}_{ int} }) ([\eta ]\psi \in x) &{}\quad \text{ Lemma } \text{39 } \\ &{}\quad \text{ iff }&{}\quad (\forall \eta \in {\mathcal {L}}_{{ PAL}_{ int} }) (x, \theta ^c)\models [\eta ]\psi &{}\quad \text{(IH) } \\ &{}\quad \text{ iff }&{}\quad (x, \theta ^c)\models \Box \psi &{}\quad \text{ semantics } \\ \end{array} \end{aligned}$$

#### Theorem 41

$${ APAL}_{ int} $$ is complete with respect to the class of all topo-models.

#### Proof

Let $$\varphi \in {\mathcal {L}}_{{ APAL}_{ int} }$$ such that $$\not \vdash \varphi $$, i.e., $$\varphi \not \in { APAL}_{ int} $$ (Recall that $${ APAL}_{ int} $$ is the smallest theory). Then, by Lemma 36, $${ APAL}_{ int} +\lnot \varphi $$ is a consistent theory and, by Lemma 35.1, $$\lnot \varphi \in { APAL}_{ int} +\lnot \varphi $$. By Lemma 37, the consistent theory $${ APAL}_{ int} +\lnot \varphi $$ can be extended to a maximally consistent theory *y* such that $${ APAL}_{ int} +\lnot \varphi \subseteq y$$. Since *y* is maximally consistent and $$\lnot \varphi \in y$$, we obtain $$\varphi \not \in y$$ (by Proposition 33). Then, by Lemma 40 (Truth Lemma), $${\mathcal {X}}^c, (y, \theta ^c)\not \models \varphi $$. $$\square $$

## S4 knowledge on multi-agent topo-models

In the literature of epistemic logic, not only *S*5 but also weaker systems such as *S*4 (Hintikka 1962), *S*4.2 (Lenzen 1978; Stalnaker 2006), and *S*4.3 (van der Hoek 1991; Baltag and Smets 2008) have commonly been studied as epistemic logics for agents with different reasoning powers. Among the aforementioned systems, *S*4 especially is of topological importance since it has been proven (due to McKinsey and Tarski 1944 in a different but still topological setting, where the knowledge modality is interpreted as the interior operator) that *S*4 is the logic of all topological spaces. In this section we propose a weaker topological semantics for the language ${\mathcal {L}}_{{ APAL}_{ int} }$ making only *S*4 axioms for knowledge sound, rather than the *S*5 axioms. This way we show that our multi-agent topo-models are more general than (Moss and Parikh 1992; Dabrowski et al. 1996; Bjorndahl 2017; Wáng and Ågotnes 2013a), in the sense that they can be adapted to model this weaker notion of knowledge, namely *S*4 type of knowledge. This result further suggests that we might be able to model intermediate knowledge notions such as *S*4.2 and *S*4.3 type knowledge on similar structures and poses the question of identifying such structures, which we aim to pursue in future work.

The *S*4 type of knowledge does not satisfy the axiom *K*-5: $$\lnot K_i\varphi \rightarrow K_i\lnot K_i\lnot \varphi $$, and the topo-models on which it is interpreted are therefore also different. We define the logic $$w{ EL}_{ int} $$ interpreted on *weak topo-models*, its axiomatization, and corresponding extensions to $$w{ PAL}_{ int} $$ and $$w{ APAL}_{ int} $$.

### Definition 42

A *weak multi-agent topological model* (weak topo-model) is a topo-model ${\mathcal {M}}=(X, \tau , \Phi , V)$ as in Def. 10 with clause 3 replaced by

3. for all $y\in X$, if $y\in \theta (x)(i)$ then $y\in {\mathcal {D}}(\theta )$ and $\theta (y)(i)\subseteq \theta (x)(i)$.

A weak topo-frame is defined analogously to Definition 11.

### Definition 43

The axiomatization of $$w{ EL}_{ int} $$ is that of $${ EL}_{ int} $$ minus the axiom *K*-5. The axiomatizations for $$w{ PAL}_{ int} $$ and $$w{ APAL}_{ int} $$ are the obvious further extensions with the $$*$$ and $$**$$-ed axioms. (See Table 1).

Soundness of $$w{ EL}_{ int} , w{ PAL}_{ int} $$, and $$w{ APAL}_{ int} $$ follow from Proposition 23 and Corollary 24. As for completeness, we again use a canonical model construction similar to the one for the stronger logics, however, adapted for the *S*4-type knowledge. Let us first introduce some notation and basic concepts.

Let $$X^c$$ be the set of all maximally consistent sets of $$w{ EL}_{ int} $$, where a maximally consistent set of $$w{ EL}_{ int} $$ is defined similarly as in Sect. 4.1. We define relations $$R^c_i$$ on $$X^c$$ as

$$\begin{aligned} xR^c_i y \ \text{ iff } \ \forall \varphi \in \mathcal {L}_{{ EL}_{ int} }(K_i\varphi \in x \ \text{ implies } \ \varphi \in y). \end{aligned}$$

Let $R^c_i(x)$ denote the upward closed set generated by *x* with respect to the relation $R^c_i$, i.e., $R^c_i(x)=\{y\in X^c \ | \ xR^c_i y\}$. Moreover, we define $\widehat{\varphi }=\{y\in X^c \ | \ \varphi \in y\}$. Observe that $x\in \widehat{\varphi }$ iff $\varphi \in x$.

### Definition 44

We define the (weak) canonical model ${\mathcal {X}}^c=(X^c, \tau ^c, \Phi ^c, V^c)$ as follows:

- $$X^c$$ is the set of all maximally consistent sets of $$w{ EL}_{ int} $$;
- $\tau ^c$ is the topological space generated by the subbase
  $$\begin{aligned} \Sigma =\{R^c_i(x)\cap \widehat{{ int} (\varphi ) } \ | \ x\in X^c, \varphi \in \mathcal {L}_{{ EL}_{ int} }\quad \text{ and }\quad i\in {\mathcal {A}}\}; \end{aligned}$$
- $$x\in V^c(p) \ \text{ iff } \ p\in x, \ \text{ for } \text{ all } \ p\in Prop $$;
- $\Phi ^c=\{\theta ^c|_U\;|\;U\in \tau ^c\}$, where we define $\theta ^c:X^c\rightarrow {\mathcal {A}}\rightarrow \tau ^c$ as $\theta ^c(x)(i)=R^c_i(x)$, for $x\in X^c$ and $i\in {\mathcal {A}}$.

Observe that $(X^c, \tau ^c, \Phi ^c)$ is a weak topo-frame. This can be shown as in the proof of Lemma 28. As in the previous case we have $\widehat{{ int} (\top ) } =X^c$, thus, each $R^c_i(x)$ is an open set in $\tau ^c$. Moreover, $\Phi ^c$ satisfies the required properties of the elements of $\Phi$ given in Definition 42. Observe that ${\mathcal {D}}(\theta ^c)=X^c$ and ${\mathcal {D}}(\theta ^c|_U)= U$ for all $U\in \tau ^c$. Moreover, $\theta ^c|_U(x)(i)=R^c_i(x)\cap U$ when $x\in U$.

### Lemma 45

(Truth Lemma) For every $\varphi \in \mathcal {L}_{{ EL}_{ int} }$ and for each $x\in X^c$

$$\begin{aligned} \varphi \in x\quad \text{ iff }\quad {\mathcal {X}}^c, (x, \theta ^c)\models \varphi . \end{aligned}$$

### Proof

Proof is similar to the proof of Lemma 29 except that that we replace each $[x]_i$ by $R_i^c(x)$. $\square$

### Theorem 46

$$w{ EL}_{ int} $$, $$w{ PAL}_{ int} $$, and $$w{ APAL}_{ int} $$ are complete with respect to the class of all weak topo-models.

### Proof

For completeness of $$w{ EL}_{ int} $$, let $$\varphi \in {\mathcal {L}}_{{ EL}_{ int} }$$ such that $$w{ EL}_{ int} \not \vdash \varphi $$. This implies that $$\{\lnot \varphi \}$$ is a consistent set. Then, by Lindenbaum’s Lemma, it can be extended to a maximally consistent set *x* such that $$\lnot \varphi \in x$$. Therefore, by Truth Lemma 2 (Lemma 45), $$X^c, (x, \theta ^c)\not \models \varphi $$. For completeness of $$w{ PAL}_{ int} $$, see proof of Theorem 31. The completeness proof of $$ { APAL}_{ int} $$ follows similarly as in Theorem 41, however, the canonical model is the same as for $$w{ EL}_{ int} $$, except that the maximally consistent sets are maximally consistent theories of $$w{ APAL}_{ int} $$.

We therefore obtain that the semantic behaviour of the epistemic modality $K_i$ in our setting depends on the properties of the neighbourhood functions similar to the case for the standard neighbourhood semantics (see, e.g., Chellas 1980) rather than the subset space setting where *S*5 type of knowledge seems intrinsic to the semantics. Moreover, by appropriate modifications on condition (3) of Definition 42, we can generalize our setting further to work with knowledge modalities of intermediate strength, such as *S*4.2 and *S*4.3 type of knowledge. If we add further conditions to Definition 42, we obtain *S*4.2 and *S*4.3 types of knowledge. More precisely, for *S*4.2 we add

- $3'.$ for all $y, z\in X$, if $y, z\in \theta (x)(i)$ then $y, z\in {\mathcal {D}}(\theta )$ and $\theta (y)(i)\cap \theta (z)(i)\not =\emptyset$ and for *S*4.3 we add
- $3'.$ for all $y, z\in X$, if $y, z\in \theta (x)(i)$ then $y, z\in {\mathcal {D}}(\theta )$ and either $\theta (y)(i)\subseteq \theta (z)(i)$ or $\theta (z)(i)\subseteq \theta (y)(i)$.

Soundness and completeness results for these cases follow similarly as in the above case.

On the other hand, it is not trivial whether and how our semantics can be adapted to versions of $$w{ EL}_{ int} $$ in which the modalities $$K_i$$ are weaker than *S*4. It is not hard to see that we can obtain such semantics that makes *KT*-, and even *K*-type modalities sound, by simply dropping the conditions (3) and also (1) of Definition 42, respectively. However, it is an open question whether these systems are complete with respect to the corresponding semantics. Roughly speaking, in the current setting, what also makes $$K_i$$ a topological modality that interacts well with the interior operator is it being at least an *S*4 type modality (see, e.g., van Benthem and Bezhanishvili 2007, Section 2 for this connection). A closer look at the canonical model constructions and the corresponding truth lemmas (Lemmas 29, 40 and 45) reveals that it is crucial in the completeness proofs that the canonical relations for the $$K_i$$ modalities are reflexive and transitive. Topological semantics for these weaker modalities is left for future research.

## Comparison to other work

In this section we compare our work in greater detail to some of the prior literature that we already referred to. In this comparison, a justified large position is taken by an embedding from single-agent topological semantics to multi-agent topological semantics and vice versa, wherein the (single-agent) work of Bjorndahl (2017) plays a large role. His use of the interior operator and topological semantics motivated our own approach: our semantics for ${\mathcal {L}}_{{ EL}_{ int} }$ and ${\mathcal {L}}_{{ PAL}_{ int} }$ are essentially multi-agent extensions of Bjorndahl’s semantics for the single-agent versions of these languages. This is the first subsection. The subsection after that contains a review of other related works.

### From multi-agent to single-agent and vice versa

We show that we can construct a point-wise modally equivalent topo-model $(X, \tau , \Phi , V)$ from a topological model without functions $(X, \tau , V)$ as in Bjorndahl (2017) and vice versa. To recall, Bjorndahl (2017) uses subset space models based on topological spaces, i.e., his models are the same as our topological models without functions; denoted by ${\mathfrak {M}}=(X, \tau , V)$. Moreover, just as in the standard subset space semantics (Moss and Parikh 1992; Parikh et al. 2007), he evaluates the formulas with respect to pairs of the form (*x*, *U*) where $x\in U\in \tau$. The crucial modalities $K\varphi$, ${ int} (\varphi )$ and $[\psi ]\varphi$ are therefore interpreted as

$$\begin{aligned} \begin{array}{lll} (x, U)\models K\varphi &{}\quad \text{ iff } &{}\quad (\forall y \in U)((y, U)\models \varphi )\\ (x, U)\models { int} (\varphi ) &{}\quad \text{ iff }&{}\quad x\in { Int} [\![ \varphi ]\!]^U \\ (x,U)\models [\psi ]\varphi &{}\quad \text{ iff } &{}\quad (x, U)\models { int} (\varphi ) \ \text{ implies } \ (x, { Int} ([\![ \varphi ]\!]^U) )\models \psi \end{array} \end{aligned}$$

where $$p\in \mathrm {Prop}$$, and $$[\![ \varphi ]\!]^U=\{ y\in U \ | \ (y, U)\models \varphi \}$$.

In the single-agent case, it is clear that a neighbourhood situation $(x,\theta )$ of a given topo-model ${\mathcal {M}}=(X, \tau , \Phi , V)$ reverts to an epistemic scenario (*x*, *U*) of ${\mathcal {M}}^- =(X, \tau , V)$ as in Bjorndahl (2017) and van Ditmarsch et al. (2014), where ${\mathcal {M}}^-$ denotes ${\mathcal {M}}=(X, \tau , \Phi , V)$ without the $\Phi$ component and $U = \theta (x)(i)$ (and where *i* is the unique agent, i.e., ${\mathcal {A}}=\{i\}$). For the other direction, given a model (without a neighbourhood function set) ${\mathfrak {M}}=(X, \tau , V)$, for each epistemic scenario $(x, U)\in {\mathfrak {M}}$, we define a neighbourhood function $\theta _U: X\rightharpoonup \{i\}\rightarrow \tau$ such that ${\mathcal {D}}(\theta _U)=U$ and $\theta (x)( i)=U$ for all $x\in U$. We therefore define the neighbourhood function set for ${\mathfrak {M}}$ as

$$\begin{aligned} \Phi _{{\mathfrak {M}}}=\{\theta _U \ | \ (x, U)\in {\mathfrak {M}}\}, \end{aligned}$$

where $\Phi _{{\mathfrak {M}}}$ denotes the neighbourhood function set constructed from ${\mathfrak {M}}$ in the above described way. It is not hard to see that $\Phi _{{\mathfrak {M}}}$ satisfies the properties given in Definition 11, and thus it is indeed a neighbourhood function set on the underlying topological space of ${\mathfrak {M}}$. Therefore, ${\mathfrak {M}}^+= (X, \tau , \Phi _{{\mathfrak {M}}}, V)$ is a topo-model given any ${\mathfrak {M}}=(X, \tau , V)$.

#### Theorem 47

1. For any ${\mathfrak {M}}=(X, \tau , V)$, any $(x, U)\in {\mathfrak {M}}$ and any $\varphi \in {\mathcal {L}}_{{ PAL}_{ int} }$,
  $$\begin{aligned} {\mathfrak {M}}, (x, U)\models \varphi \quad \text{ iff }\quad {\mathfrak {M}}^+, (x, \theta _U)\models \varphi . \end{aligned}$$
2. For any ${\mathcal {M}}=(X, \tau , \Phi , V)$, any $(x, \theta )\in {\mathcal {M}}$, and any $\varphi \in {\mathcal {L}}_{{ PAL}_{ int} }$,
  $$\begin{aligned} {\mathcal {M}}, (x, \theta )\models \varphi \quad \text{ iff }\quad {\mathcal {M}}^-, (x, \theta (x)(i))\models \varphi . \end{aligned}$$

#### Proof

The proofs for both items follow in a similar way by induction on the size of the formulas in $${\mathcal {L}}_{{ PAL}_{ int} }$$: using the size measure $$S(\varphi )$$ from Definition 3 provides the desired result via Lemma 7. The cases for the propositional variables, Booleans and the modalities *K* and $${ int} $$ are standard. The case $$\varphi :{=}[\psi ]\chi $$ for the public announcement modality follows by subinduction on $$\chi $$. It is crucial for this case that our semantics and Bjorndahl’s semantics make the same reduction axiom schemes, namely the axiom schemes (R1)–(R6) given in Table 1, valid. Here we present only the subcase for $$\chi =p$$ of item (1). The other cases follow in a similar way.

**Subcase**$$\varphi :{=} [\psi ]p$$

$$\begin{aligned} \begin{array}{llll} {\mathfrak {M}}, (x, U)\models [\psi ]p &{}\quad \text{ iff } &{}\quad {\mathfrak {M}}, (x, U)\models { int} (\psi ) \rightarrow p &{}\quad \text{ by } \text{ the } \text{ validity } \text{(R1)* } \\ &{}\quad \text{ iff } &{}\quad {\mathfrak {M}}^+, (x, \theta _U)\models { int} (\psi ) \rightarrow p &{}\quad \text{ by } \text{ Lemma } \text{7.1 } \text{ and } \text{(IH) }\\ &{}\quad \text{ iff } &{}\quad {\mathfrak {M}}^+, (x, \theta _U)\models [\psi ]p &{}\quad \text{ by } \text{ the } \text{ validity } \text{(R1)** } \\ \end{array} \end{aligned}$$

*: with respect to the semantics in Bjorndahl (2017)

**: with respect to the semantics given in Definition 13. $\square$

In other words, Theorem 47.1 states that ${\mathfrak {M}}, (x, U)$ and ${\mathfrak {M}}^+, (x, \theta _U)$ are modally equivalent with respect to ${\mathcal {L}}_{{ PAL}_{ int} }$. Moreover, for all $\varphi \in {\mathcal {L}}_{{ PAL}_{ int} }$, ${\mathfrak {M}}\models \varphi \ \text{ iff } \ {\mathfrak {M}}^+\models \varphi$, i.e., ${\mathfrak {M}}$ and ${\mathfrak {M}}^+$ are (globally) modally equivalent with respect to the same language. Further, Theorem 47.2 shows that ${\mathcal {M}}, (x, \theta )$ and ${\mathcal {M}}^-, (x, \theta (x)(i))$ are modally equivalent with respect to ${\mathcal {L}}_{{ PAL}_{ int} }$. However, ${\mathcal {M}}$ is not necessarily (globally) modally equivalent to ${\mathcal {M}}^-$, as the following example demonstrates.

#### Example 48

The reason why ${\mathcal {M}}$ and ${\mathcal {M}}^-$ are not necessarily modally equivalent is that while ${\mathcal {M}}^-$ reverts to using the full topology $\tau$, the view on that in ${\mathcal {M}}$ is restricted by $\Phi$. For a counterexample, consider the topo-model ${\mathcal {M}}=(X, \tau , \Phi , V)$ where $X=\{1, 2\}$ and $\tau$ is the discrete topology on *X*. We set $\Phi =\{\theta \}$ where ${\mathcal {D}}(\theta )=\{2\}$ and $\theta (2)=\{2\}$. Hence, the only neighbourhood situation of ${\mathcal {M}}$ is $(2, \theta )$. Finally we let $V(p)=\{1\}$. Therefore, ${\mathcal {M}}, (2, \theta )\models \lnot K p$ and as $(2, \theta )$ is the only neighbourhood situation of the model, we obtain ${\mathcal {M}}\models \lnot K p$ of item. On the other hand, $(1, \{1\})$ is an epistemic scenario of ${\mathcal {M}}^-$ and ${\mathcal {M}}^-, (1, \{1\})\models K p$, therefore, ${\mathcal {M}}^-\not \models \lnot K p$.

Moreover, as demonstrated in Sect. 6.1, the single-agent version of our proposal does not lead to any restriction compared to Bjorndahl (2017) and even provides a larger class of models to work with.

### Survey of the literature

In this section, we compare mainly three aspects of our work to that of others in the relevant literature:

**Multi-agent epistemic systems.** Multi-agent epistemic systems with subset space-like semantics have been proposed in Heinemann (2008, 2010), Başkent (2007) and Wáng and Ågotnes (2013a), however, none of these are concerned with public or arbitrary public announcements. An unorthodox approach to multi-agent knowledge is proposed in Heinemann (2008, 2010). Roughly speaking, instead of having a knowledge modality $K_i$ for each agent as a primitive operator in his syntax, Heinemann uses additional operators to define $K_i$ and his semantics only validates the $\mathsf {S4}$-axioms for $K_i$. The necessitation rule for $K_i$ does not preserve validity under the proposed semantics (Heinemann 2008, 2010). On the other hand, we follow the methods of dynamic epistemic logic in our multi-agent generalization by extending the single-agent case with a knowledge modality $K_i$ for each agent and propose a multi-agent topological semantics for this language general enough to model both $\mathsf {S4}$ and $\mathsf {S5}$ types of knowledge, and flexible enough for further generalizations as shown in Sect. 5. Another multi-agent logic of subset spaces is developed in Wáng and Ågotnes (2013a). This setting uses multi-agent versions of both knowledge $K_i$ and effort $\Box _i$, where, for example, $\Diamond _1 K_2p$ is read as “agent 1 comes up with evidence so that agent 2 gets to know *p*” (Wáng and Ågotnes 2013a, p. 1160). They have left the question of how to model an agent-independent effort operator open, while pointing out its connection to the arbitrary announcement modality of Balbiani et al. (2008). Besides, no announcements or further generalizations (unlike in their other, single-agent, work Wáng and Ågotnes 2013b) are considered in Wáng and Ågotnes (2013a), and a purely topological case is left for future research. To this end, we believe our work at least partially answers some of their open questions. Their use of partitions for each agent instead of a single neighbourhood is compatible with our requirement that all neighbourhoods for a given agent be disjoint. A further difference from the existing literature is that we restrict our attention to topological spaces and prove our results by means of topological tools. For example, our completeness proofs employ direct topological canonical model constructions without a detour referring to different types of semantics and completeness results therein.

**Completeness proof.** We applied the new completeness proof for arbitrary public announcement logic of Balbiani and van Ditmarsch (2015) to a topological setting. The modality $${ int} $$ in our system demands a different complexity measure in the Truth Lemma of the completeness proof of $${ APAL}_{ int} $$ than in Balbiani and van Ditmarsch (2015). Moreoever, we modified the complexity measure given in van Ditmarsch et al. (2015b) to make it work for both the completeness of $${ APAL}_{ int} $$ and of $${ PAL}_{ int} $$. The canonical modal construction is as in Bjorndahl (2017) with some multi-agent modifications. We defined the set $$\Sigma $$ from which the topology of the canonical model is generated in a similar way as in Bjorndahl (2017), however, having multiple agents renders this set weaker in the sense that while it constitutes a base in the single-agent case, it becomes a subbase in the multi-agent setting.

**Single agent case.** In standard (single-agent) subset space semantics (Moss and Parikh 1992; Dabrowski et al. 1996) and in the later extensions (Wáng and Ågotnes 2013b; Bjorndahl 2017; Balbiani et al. 2013; van Ditmarsch et al. 2014), the modality *K* quantifies over the elements of a given open neighbourhood *U* that is fixed from the beginning of the evaluation. This makes *K* behave like a universal modality within *U*, therefore, *S*5 as an underlying epistemic system becomes intrinsic to the semantics. However, in our proposal, the soundness of the epistemic axioms (i.e., axioms involving only the modality *K*) depends on the constraints posed on the neighbourhood functions and relaxing these constraints enables us to work with weaker notions of knowledge, such as *S*4 as shown in Sect. 5. In this sense, our approach generalizes the epistemic aspect of aforementioned literature. Moreover, Balbiani et al. (2013) proposed subset space semantics for arbitrary announcements, however, their approach does not go beyond the single-agent case and the semantics provided is in terms of model restriction.

**Temporal epistemic logics and protocol logics.** We can compare the logics we have presented here to some other dynamic modal logics. When modelling dynamic operators, there are two main approaches: one can either start with an initial model and then view operators as *changing* or *transforming* the initial model, or one can include the dynamic operators in the initial model, and view operators as *transitions* within this unchanging model. In our logics we take the second approach: the dynamic operators are not model transformers but are interpreted by a shift of perspective in the unmodified initial model, represented by an updated neighbourhood function. Our dynamic systems $${ APAL}_{{ int} }$$ and $${ PAL}_{{ int} }$$ are therefore in contrast with the framework of traditional dynamic epistemic logic; in fact they are also akin to, for example, temporal epistemic logics (Halpern et al. 2004; van der Meyden and Wong 2003) or dynamic (PDL-type) logics (Harel et al. 2000), and more specifically to the subset logic for reasoning about change (Georgatos 2011). In such logics temporal/action operators are also interpreted by a perspective shift, i.e., by a relation within the model representing such modalities. For example, in PDL-style, if *x* is a world, then $$\mathcal {M}, x \models [a]p$$ if and only if $$(x, y)\in R_a$$ implies $$\mathcal {M}, y \models p$$. A similar PDL-stype approach is adapted to a subset space logic by Georgatos (2011), and he studied an action-based knowledge change in this framework. In temporal epistemic logics the interaction is slightly more involved, so we have, for example, that when $$x_1x_2x_3$$ is a path, $$\mathcal {M}, x_1x_2x_3 \models X p$$ if $$\mathcal {M}, x_2x_3 \models p$$, involving a similar shift of perspective or state, rather than a model transformation. In $${ APAL}_{{ int} }$$, our ‘designated points’ are pairs $$(x,\theta )$$ which we can see as a multi-agent generalization (viewed as, $$(x,\theta (x)(a),\theta (x)(b))$$) of the pairs (*x*, *U*) in subset space logic. One difference from the PDL and temporal operator interpretation is that the perspective shift for public announcement interpretation is in the second argument of the pair, the neighbourhood function, rather than in the first argument, which represents the agent’s actual state: $$\mathcal {M}, (x,\theta ) \models [q] p$$ iff $$\mathcal {M}, (x,\theta ) \models { int} (q) $$ implies $$\mathcal {M}, (x,\theta ^q) \models p$$. Another difference from many temporal logics is that subject to executability, the public announcement is a ‘computable’ dynamic modality: although it is a perspective shift, the shift is *computed* from the announcement formula and not a given in the underlying model, as in LTL or CTL, where we have maximum freedom to specify the underlying model. However, the executability precondition $${ int} $$ is also reminiscent of other logics, namely (dynamic epistemic, or dynamic) logics of protocols, see e.g. Wang (2010) and Hoshi (2009). In such logics, public announcements, or other epistemic actions, cannot be executed merely if the announcement formula is true, but only if the announcement formula is in the list of ‘permitted formulas to be announced’, i.e., allowed according to the *protocol*. A strong link between logics of protocols, dynamic epistemic logics, and temporal epistemic logics is provided in works van Benthem et al. (2009) and Dégremont et al. (2011): instead of, as in dynamic epistemic logic, thinking of an initial model that is transformed by successive dynamic modalities (such as for announcement), we can also see these dynamic transitions as interpreted by internal shifts in a larger model, namely the so-called protocol-generated tree that consists of the initial model plus the transformed model(s) *in relation*. To illustrate this, for a final example, given $$\mathcal {M}, x \models [q] p$$ in standard public announcement logic, instead of interpreting the announcement of *q* as a model restriction, i.e., as $$\mathcal {M}|q, x \models p$$, we can also consider the disjoint union of $$\mathcal {M}$$ and $$\mathcal {M}|q$$ plus a pair $$(x_0,x_1)$$ linking them, and see the interpretation of [*q*] as a shift along $$(x_0,x_1)$$ in $$\mathcal {M} \oplus \mathcal {M}|q$$. This is instructive, because here we see again that, unlike in $${ APAL}_{{ int} }$$, the shift can be seen as occurring in an accessibility relation, and does not have to consist of shrinking a neighbourhood from $$(x,\theta )$$ to $$(x,\theta ^p)$$.

## Conclusions

We have proposed a multi-agent topological semantics for knowledge, public and arbitrary announcements in the style of subset space semantics. In particular we provided a multi-agent semantic framework, based on topological spaces, that eliminates the so-called problem of “jumping out of the epistemic range” in the evaluation of higher-order knowledge formulas involving different agents. In our setup all agents have the same observational power in the sense that they have access to exactly the same collection of potential evidence, represented by each topo-model carrying only one topology. In order to model the informational attitudes of a group of agents with different observational powers, one could associate a possibly different topology with each agent together with a “common” topology representing all potential evidence. Moreover, the studied notions of dynamics of learning something brought about by announcements were of public nature, and the information source was assumed to be external. van Ditmarsch et al. (2017) generalizes the topological public announcement semantics of this chapter for semi-private announcement, again assuming the information source to be external. We can further generalize our setting to an arbitrary *epistemic action* logic.

Our goal was not so much to provide a multi-agent generalization of SSL or topologic per se, but to have an intuitively appealing interpretation of the *effort-like* modality $\Box$ (information change brought about by any announcement) in a multi-agent setting, by way of modelling it as “open-set shrinking”. In this respect, our work complements (Georgatos 2011; Bjorndahl 2017), and can be seen as a step toward discovering the interplay between dynamic epistemic logic and topological reasoning.

Unsurprisingly, working with $\mathsf {S5}$-type of knowledge required a partitioning of the (sub)domain of a topological space. This might seem like a restrictive requirement since it rules out working with more familiar spaces such as the natural topology of open intervals on the real line or the Euclidean space. However, as long as multiple $\mathsf {S5}$-type agents are concerned, we believe it is hard to avoid such a restriction, if it is possible at all. We then axiomatized the multi-agent logic of observation-based knowledge ${ EL}_{{ int} }$, its extension with public announcements ${ PAL}_{{ int} }$, and also with arbitrary public announcements ${ APAL}_{{ int} }$. The arbitrary announcement modality $\Box \varphi$ capturing “stability of the truth of $\varphi$ after any announcement” comes close to the intuition behind the effort modality $\Box \varphi$ as “stability of the truth of $\varphi$ after any effort”. In Baltag et al. (2017), these two modalities are even proven to be equivalent in the single-agent setting. However, the appropriate interpretation of effort in the multi-agent setting and its connection to the arbitrary announcement modality still remain elusive. The connection between the effort modality and the arbitrary announcement modality has also been observed in Wáng and Ågotnes (2013a), however, providing a formal analysis regarding the link between these two modalities in a multi-agent setting is not straightforward: there is not yet agreement on how to interpret the effort modality in a multi-agent framework (see Sect. 6 for a comparison with other work on multi-agent subset space semantics). The existing proposals neither agree on the general framework, nor are they entirely compatible with one another or with our multi-agent topological setting. We leave this question as future work.

We are currently investigating expressivity and (un)decidability. If the logic ${ APAL}_{{ int} }$ is decidable, this would contrast nicely with the undecidability of arbitrary public announcement logic (French and van Ditmarsch 2008). Otherwise, there may be decidable fragments when restricting the class of models to particular topologies.

## Footnotes

1 The topological version of the subset space logics, the so-called *topologic*, has been extensively studied by Georgatos (1993, 1994, 1997).

2 The epistemic use of topological spaces in the same spirit as information structures can be traced back to the 1930s and 1940s, where topological spaces served as models for intuitionistic languages, and open sets are considered to be ‘pieces of evidence’, ‘observable properties’ concerning the actual state (see, e.g., Troelstra and van Dalen 1988). This interpretation assigned to open sets constitutes the basic epistemic motivation behind our use of topological models in this paper as well. Variations of this idea can also be found in domain theory in computer science (Abramsky 1987, 1991; Vickers 1989), and guide the research program of “topological” formal learning theory initiated by Kelly (1996), Schulte and Juhl (1996), Kelly et al. (1995), Kelly and Lin (2011), Baltag et al. (2015) in formal epistemology.

3 The axioms (R3) and (R4) are in fact derivable from the other axioms and rules in $\mathbf {PAL}_{ int}$, and in $\mathbf {APAL}_{ int}$. Here we prefer to use the most standard axiomatizations.
